# Genomics of NSCLC patients both affirm PD-L1 expression and predict their clinical responses to anti-PD-1 immunotherapy

**DOI:** 10.1186/s12885-018-4134-y

**Published:** 2018-02-27

**Authors:** Kim A. Brogden, Deepak Parashar, Andrea R. Hallier, Terry Braun, Fang Qian, Naiyer A. Rizvi, Aaron D. Bossler, Mohammed M. Milhem, Timothy A. Chan, Taher Abbasi, Shireen Vali

**Affiliations:** 1 Iowa Institute for Oral Health Research, College of Dentistry, The University of Iowa, 801 Newton Road, Iowa City, IA 52242 USA; 2 Cellworks Research India Ltd., Whitefield, Bangalore, 560066 India; 3 Biomedical Engineering, The University of Iowa, 5318 SC, Iowa City, IA 52242 USA; 4 Division of Biostatistics and Research Design, College of Dentistry, The University of Iowa, 801 Newton Road, Iowa City, IA 52242 USA; 5 Division of Hematology/Oncology, Columbia University Medical Center, 177 Fort Washington Avenue, New York, NY 10032 USA; 6 Molecular Pathology Laboratory, Department of Pathology, University of Iowa Hospitals and Clinics, 200 Hawkins Dr., C606GH, Iowa City, IA 52242 USA; 7 Clinical Services, Experimental Therapeutics, Melanoma and Sarcoma Program, Holden Comprehensive Cancer Center, The University of Iowa, Iowa City, IA 52242 USA; 8 Department of Radiation Oncology, Human Oncology and Pathogenesis Program, Immunogenomics and Precision Oncology Platform, Memorial Sloan Kettering Cancer Center, New York, NY 10065 USA; 9 Cellworks Group, Inc., 2033 Gateway Place Suite 500, San Jose, CA 95110 USA

**Keywords:** Computational modeling, PD-1, PD-L1, NSCLC, Immunotherapy

## Abstract

**Background:**

Programmed Death Ligand 1 (PD-L1) is a co-stimulatory and immune checkpoint protein. PD-L1 expression in non-small cell lung cancers (NSCLC) is a hallmark of adaptive resistance and its expression is often used to predict the outcome of Programmed Death 1 (PD-1) and PD-L1 immunotherapy treatments. However, clinical benefits do not occur in all patients and new approaches are needed to assist in selecting patients for PD-1 or PD-L1 immunotherapies. Here, we hypothesized that patient tumor cell genomics influenced cell signaling and expression of PD-L1, chemokines, and immunosuppressive molecules and these profiles could be used to predict patient clinical responses.

**Methods:**

We used a recent dataset from NSCLC patients treated with pembrolizumab. Deleterious gene mutational profiles in patient exomes were identified and annotated into a cancer network to create NSCLC patient-specific predictive computational simulation models. Validation checks were performed on the cancer network, simulation model predictions, and PD-1 match rates between patient-specific predicted and clinical responses.

**Results:**

Expression profiles of these 24 chemokines and immunosuppressive molecules were used to identify patients who would or would not respond to PD-1 immunotherapy. PD-L1 expression alone was not sufficient to predict which patients would or would not respond to PD-1 immunotherapy. Adding chemokine and immunosuppressive molecule expression profiles allowed patient models to achieve a greater than 85.0% predictive correlation among predicted and reported patient clinical responses.

**Conclusions:**

Our results suggested that chemokine and immunosuppressive molecule expression profiles can be used to accurately predict clinical responses thus differentiating among patients who would and would not benefit from PD-1 or PD-L1 immunotherapies.

**Electronic supplementary material:**

The online version of this article (10.1186/s12885-018-4134-y) contains supplementary material, which is available to authorized users.

## Background

In clinical trials of PD-1 or PD-L1 checkpoint immunotherapies, patients with NSCLC separate into groups that respond or do not respond to immunotherapy treatment [1–4]. Objective responses for NSCLC in these studies range from 19.0–23.0%. Patients are selected for immunotherapy based on immunohistochemistry (IHC) detection of PD-L1 reactivity. Positive PD-L1 reactivity in tumors is considered to be important to predicting the success of PD-1 and PD-L1 immunotherapy treatments [2, 5]. However, IHC results to detect PD-L1 reactivity can vary depending upon different IHC platforms, differences in anti-PD-L1 antibodies, differences in scoring systems, and differences in positivity cut-off values [4, 6–9]. In the Blueprint PD-L1 IHC Assay Comparison Project [10], similar antibody-specific differences were seen. In all, this variability presents challenges for using PD-L1 reactivity as a sole marker for diagnosis and as a marker to predict the success of PD-1 and PD-L1 immunotherapy treatments.

More likely, there is a complex profile of molecules that contributes to the regulation of PD-L1 and to the subsequent immunosuppressive effects that NSCLC cells have on immune cells [11]. Chae et al. suggested that reliable predictive molecules need to be identified that can be used to select patients who would benefit from immunotherapy, yet limit the exposure of patients who would not benefit or have adverse reactions [12]. Multifaceted predictive biomarker systems have also been proposed that contain input on PD-L1 expression, tumor mutations, and the roles of inflammatory cells to identify patients that would respond or not respond to immunotherapy treatment [13, 14]. For better treatment outcomes, there is a recognized need to develop additional methods that can identify a profile of molecules that contributes to the regulation of PD-L1 expression and affirms IHC PD-L1 positive reactivity. One approach is to use the influence of patient cell genomics on tumor cell signaling to identify the downstream effects on PD-L1 expression.

In this study, we hypothesized that patient tumor cell genomics influences cell signaling and the expression of PD-L1, chemokines, and immunosuppressive molecules. We also hypothesized that these profiles can be used to predict patient clinical responses. Rizvi et al. assessed the mutational profiles that determined sensitivity to PD-L1 blockade from patients with NSCLC treated with pembrolizumab [15] and we used the Rizvi et al. dataset to test our hypothesis. We first assessed the effect of patient genomics on the expression profile of 24 molecules: PD-L1, 9 chemokines, and 14 immunosuppressive molecules. Differences among patient-specific models reflected the input that their deleterious gene mutation profiles had on modeled signaling pathways and the expression of PD-L1, chemokines, and immunosuppressive molecules. Second, we used the expression profiles of these 24 chemokines and immunosuppressive molecules to sort patients into those that would or would not respond to PD-1 immunotherapy. The 9 chemokines were used to generate an index to predict dendritic cell infiltration and PD-L1 and the 14 immunosuppressive molecules were selected as tumor-derived molecules with a long list of reported immunosuppressive functions (Additional file 1: Table S1). Our results suggest that patient-specific chemokine and immunosuppressive molecule expression profiles can be used to accurately predict clinical responses thus differentiate among patients who would or would not respond to PD-1 immunotherapy.

## Methods

### Patient clinical characteristics and mutation profiles

This was a retrospective study and patient data, clinical characteristics, and exome sequencing information for each of 34 patients were obtained directly from Supplement Table 3 of the Rizvi et al study. study [15]. To maintain anonymity, a random string generator was used to create a new random, 6-character uppercase alpha numeric string for each patient. This blinded both the identities of the patients in this study and their link to the prior published dataset we modeled.

All patients had stage IV NSCLC and were treated at Memorial Sloan Kettering Cancer Center (*n* = 29) or the University of California at Los Angeles (*n* = 5) on protocol NCT01295827. All patients had consented to Institutional Review Board-approved protocols permitting tissue collection and sequencing by the co-authors in this study (Naiyer A. Rizvi and Timothy A. Chan). All patients initiated therapy in 2012–2013 and were treated at 10 mg/kg every 2–3 weeks. Five patients were treated at 2 mg/kg every 3 weeks. The overall response rate and progression-free survival were reported to be similar across dose and schedules. PD-L1 expression on NSCLC tumor cells and immune cells by IHC was reported and scored semi-quantitatively: ≥50.0% membranous staining was considered strong, 1–49.0% was considered weak, and < 1.0% was considered negative [15].

**Fig. 1:**
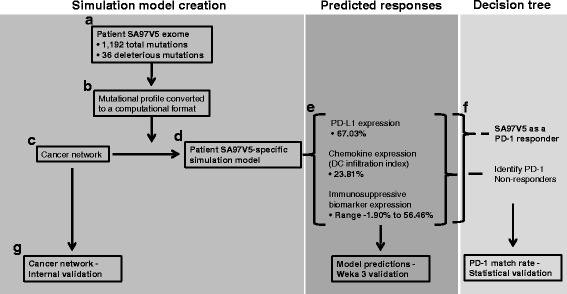
The schema for creating predictive computational simulation models to predict molecule responses and identify patients that would respond or not respond to PD-1 immunotherapy treatment using patient SA97V5 as a model example. Exome information from patient SA97V5 (**a**) contained 1192 total mutations with 36 deleterious mutations. This profile (**b**) was converted from a mutational profile to a computational format and annotated into the computational workflow to convert (**c**) a nontransformed model in the cancer network into (**d**) a patient SA97V5-specific simulation model. The patient SA97V5-specific simulation model was used to predict PD-L1 expression (e.g., 67.0% with respect to control), dendritic cell (DC) infiltration index (e.g., 23.8% with respect to control); and an immunosuppressive molecule expression profile (e.g., range − 1.9% to 56.5% with respect to controls) (**e**). Predicted expression responses were all used (**f**) to sort patients into groups that would respond or not respond to PD-1 immunotherapy treatment. SA97V5 was identified as a patient who would respond to PD-1 immunotherapy treatment. Numerous validation checks (**g**) occurred on the cancer network, the simulation model predictions, and the PD-1 match rates between the predicted responses and the patient clinical responses

Exomes from each NSCLC patient were examined using FannsDB [16], FATHMM [16], Mutation Assessor [17], Polyphen [18], PROVEAN [19], and SIFT [20]. Gene mutations deleterious to gene function were identified (Additional file 2: Table S2). For example, there were 1192 gene mutations listed for patient SA97V5 and 36 mutations were deleterious to gene function (Fig. 1).

### Simulation models

A validated cancer network containing a database of proteins involved in cell signal transduction, metabolism, and epigenetics obtained from manual review of new and published research (Additional file 3: Figure S1) was used to create patient NSCLC-specific predictive computational simulation models. This approach modeled protein-protein interactions at each step in a signaling pathway using ordinary differential equations (ODE) [21] and to predict specific pathway output [22]. Pathway protein-protein interactions at each specific node were modeled as Michaelis-Menten equations that contained the reaction, enzyme, initial concentrations of protein intermediate reactants, and parameters of the reaction like Ka, Km, kcat, Vmax, etc. ODE were solved at each step by the Radau method [23]. To demonstrate this modeling approach, an annexure section of the PD-L1 pathway is illustrated showing the step-by-step details of the protein-protein interactions at each node in the pathway as an example of the modeling process that also occured in all of the other pathways (Additional file 4: Supplementary Materials and Methods and Supplement Table 3 of the Rizvi et al study). The cancer network and the schema for creating these simulation models, predicting molecule responses, and identifying those patients who would or would not respond to PD-L1 immunotherapy is shown in Fig. 1.

NSCLC models in the cancer network were created for each patient. At the initial step, models did not contain patient-specific deleterious gene mutation profiles and were simulated to reach a homeostatic steady state, which served as the control baseline for the molecules of interest. Then patient-specific deleterious gene mutation profiles were converted into a computational format and annotated into the NSCLC cancer network, simulated to induce the patient-specific cancer disease states, and used to predict the expression of PD-L1, chemokines, and immunosuppressive molecules. At the network level, mutations of oncogenes were represented as gain of function at the activity level and mutations of tumor suppressor genes were represented as a loss of function at the activity level unless explicit functionality of the mutation was known from published studies. Copy number variations such as amplifications and deletions were represented as over-expression or deletion of gene function at the expression level. The time required to achieve a patient-specific network varied depending upon on the complexity of the patient-specific deleterious gene mutation profile.

The modeled output contained the expression profiles of 24 molecules (e.g., PD-L1, 9 chemokines, and 14 immunosuppressive molecules). PD-L1 expression was reported as percent change calculated as ((D/C)-1)*100. C was the absolute value of the non-tumorigenic baseline control (μM) and D was the absolute value of PD-L1 obtained from the patient-specific cancer state network (μM) [24]. CCL2 [25], CCL3 [26], CCL4 [27], CCL5 [28], CCL11 [29], CCL20 [30], and CX3CL1 [31] expression were determined similarly. These chemokines are capable of trafficking dendritic cells into the tumor microenvironment. Individual chemokine percent expression values were given weightage and normalized to sum to 1. A dendritic cell infiltration index was then calculated to be the sum of each prediction % change * weightage (Additional file 6: Table S4). Finally, the expression of 14 immunosuppressive molecules thought to facilitate the ability of cancer cells to escape normal tumor surveillance was determined (Additional file 1: Table S1).

Patient-specific simulation model predictions were also assessed using Weka 3, a data mining software program in Java [32]. Weka 3 contained machine learning algorithms for data pre-processing; data classification, regression, clustering, and association rules; and data visualization. Using the predicted responses in Table 1, several machine-learning algorithms were implemented to learn prediction models (Additional file 7: Table S5).

**Table 1:** Discovery (*n* = 13) and Validation (*n* = 16) datasets of patients with non-small cell lung cancer containing PD-1 clinical response and patient-specific simulation models containing PD-1 predicted response, predicted PD-L1 expression, immunosuppressive biomarker expression, and predicted dendritic cell (DC) infiltration index

DISCOVERY DATASET																			
				Immunosuppressive Biomarkers															
**Study ID**	**PD-1 Clinical Response**	**PD-1 Predicted response**	**Score**	**PDL1**	**TGFB1**	**IDO1**	**IL6**	**VEGFA**	**TDO2**	**PGE2**	**IL10**	**LGALS9**	**FASLG**	**CD47**	**CTLA4**	**PDCD1LG2**	**GM3**	**GD2**	**DC Index**
**VCMG7N**	NON-RESPONDER	NON-RESPONDER	**MATCH**	25.73	4.14	5.27	14.79	11.31	0.98	4.67	14.50	0.20	−5.99	3.20	10.76	1.36	25.57	25.57	17.85
**L11LVL**	NON-RESPONDER	NON-RESPONDER	**MATCH**	18.95	−1.08	−2.33	7.89	14.14	1.38	−7.60	8.51	−0.03	−2.32	3.74	6.15	1.51	14.48	14.48	14.73
**UC2LIA**	NON-RESPONDER	NON-RESPONDER	**MATCH**	78.62	44.67	5.44	131.81	48.07	3.35	16.14	41.95	2.68	−2.61	6.30	12.10	2.82	25.52	25.52	40.42
**X0152B**	NON-RESPONDER	NON-RESPONDER	**MATCH**	21.87	9.51	0.84	16.17	15.78	0.23	38.50	13.54	−0.43	−7.37	7.44	12.07	1.40	−0.01	−0.01	26.80
**QIA43T**	NON-RESPONDER	NON-RESPONDER	**MATCH**	41.42	57.12	−3.64	55.10	40.89	4.06	12.60	36.51	1.78	1.26	1.09	6.57	4.83	21.57	21.57	42.21
**J0T9TJ**	NON-RESPONDER	NON-RESPONDER	**MATCH**	44.40	26.90	−2.55	20.21	17.96	1.44	20.71	12.71	5.92	− 0.96	2.03	2.24	14.55	14.55	2.56	16.71
**3DJF3O**	NON-RESPONDER	NON-RESPONDER	**MATCH**	21.81	7.76	4.19	21.26	9.45	0.78	1.97	11.54	1.58	−8.74	3.34	1.97	1.72	18.63	18.63	18.29
**MG6XF2**	NON-RESPONDER	NON-RESPONDER	**MATCH**	11.08	7.54	1.97	81.93	25.47	−0.98	4.74	9.51	1.11	−1.15	2.01	17.50	0.86	5.92	5.92	5.31
**C9TGAJ**	RESPONDER	RESPONDER	**MATCH**	43.42	23.05	4.64	37.16	31.76	2.75	9.86	25.25	5.24	−11.30	6.60	11.83	4.15	40.30	40.30	37.91
**RDD2UW**	RESPONDER	RESPONDER	**MATCH**	54.86	49.36	10.35	47.04	22.34	1.97	19.41	22.51	14.47	− 0.25	3.40	2.96	4.87	29.93	29.93	32.66
**L8MTGU**	RESPONDER	RESPONDER	**MATCH**	34.59	18.21	6.31	38.22	16.49	1.36	2.37	17.06	3.48	− 8.50	4.08	7.70	2.37	26.69	26.69	23.76
**2FCOH7**	RESPONDER	NON-RESPONDER	**MISMATCH**	31.78	35.02	−7.84	28.65	63.79	4.39	−32.22	23.15	8.79	−5.90	6.16	48.00	8.42	41.16	41.16	26.35
**P90A0O**	RESPONDER	RESPONDER	**MATCH**	34.09	19.79	5.98	35.86	15.08	1.67	2.38	17.45	6.00	−7.27	4.36	3.32	2.64	29.91	29.91	28.49
		**Total score**	**92.31**																
VALIDATION DATASET																			
				Immunosuppressive Biomarkers															
**Study ID**	**Clinical Response**	**Predicted response**	**Score**	**PDL1**	**TGFB1**	**IDO1**	**IL6**	**VEGFA**	**TDO2**	**PGE2**	**IL10**	**LGALS9**	**FASLG**	**CD47**	**CTLA4**	**PDCD1LG2**	**GM3**	**GD2**	**DC Index**
**195P5D**	NON-RESPONDER	NON-RESPONDER	**MATCH**	27.60	15.20	0.97	26.76	12.90	1.33	9.08	16.51	3.54	− 8.83	5.99	1.66	45.78	25.94	25.94	26.60
**ZNT6MQ**	NON-RESPONDER	NON-RESPONDER	**MATCH**	76.54	31.90	−0.45	101.96	36.03	5.64	23.13	40.46	3.63	− 0.76	9.05	2.29	10.64	41.99	41.99	48.94
**ZX7V33**	NON-RESPONDER	NON-RESPONDER	**MATCH**	33.70	5.06	5.19	19.67	6.96	0.71	−0.39	9.31	2.49	−7.64	3.26	1.76	1.45	14.28	14.28	16.00
**F3FK2W**	NON-RESPONDER	RESPONDER	**MISMATCH**	63.86	17.70	3.87	25.27	15.83	−3.05	5.47	19.72	−4.96	− 4.56	8.16	7.10	2.98	24.03	24.03	34.14
**6QFSVV**	NON-RESPONDER	RESPONDER	**MISMATCH**	43.31	44.18	5.31	42.83	19.40	1.86	14.62	18.65	12.46	− 0.10	1.88	2.10	3.29	26.70	26.70	25.50
**IPUAS9**	NON-RESPONDER	NON-RESPONDER	**MATCH**	28.49	10.25	5.43	25.29	10.86	2.11	4.85	11.77	2.65	−1.00	3.44	7.14	1.51	13.54	13.54	15.01
**GI7AGZ**	NON-RESPONDER	RESPONDER	**MISMATCH**	142.53	96.10	8.52	114.25	69.40	4.12	39.61	49.30	25.56	0.38	5.44	12.89	12.02	67.05	67.05	64.92
**CZH5YD**	NON-RESPONDER	NON-RESPONDER	**MATCH**	76.21	17.60	8.11	96.00	30.61	3.17	7.75	28.32	9.96	−2.31	5.56	7.63	32.48	32.48	3.75	26.89
**67K46M**	NON-RESPONDER	NON-RESPONDER	**MATCH**	−8.33	3.34	−1.51	8.23	2.40	−0.63	2.74	2.07	−0.55	− 6.25	175.23	−0.45	0.83	3.79	3.79	4.20
**3HDJMG**	NON-RESPONDER	NON-RESPONDER	**MATCH**	4.42	4.70	1.12	17.12	9.89	1.35	0.08	10.07	0.06	−1.35	4.61	7.45	0.17	14.17	14.17	14.40
**26YMUF**	RESPONDER	RESPONDER	**MATCH**	29.26	9.43	5.84	24.75	10.64	2.47	5.95	13.47	2.17	−1.63	6.50	7.43	0.67	25.92	25.92	23.23
**MJXYP6**	RESPONDER	RESPONDER	**MATCH**	137.90	43.67	190.94	91.44	102.75	16.56	49.41	79.19	6.75	2.07	7.67	144.61	4.60	4.13	4.13	73.86
**M9GYO4**	RESPONDER	RESPONDER	**MATCH**	185.53	12.45	5.87	53.86	33.00	4.15	11.69	25.87	2.67	−3.16	9.09	21.21	2.04	36.09	36.09	31.90
**L6ADEL**	RESPONDER	RESPONDER	**MATCH**	37.43	14.64	4.80	31.24	18.91	1.69	−1.21	20.73	3.77	−13.77	6.07	5.03	4.41	40.54	40.54	34.94
**DFZLO2**	RESPONDER	RESPONDER	**MATCH**	60.53	122.62	−0.26	51.22	57.41	3.61	229.90	62.31	8.86	−6.31	11.09	16.77	38.08	38.08	8.48	79.85
**SA97V5**	RESPONDER	RESPONDER	**MATCH**	67.03	12.87	15.30	56.46	21.85	−0.43	20.30	20.85	1.21	−1.90	5.28	10.32	2.79	20.96	20.96	23.81
		**Total score**	**81.25**																

### Clinical response projections

**Fig. 2:**
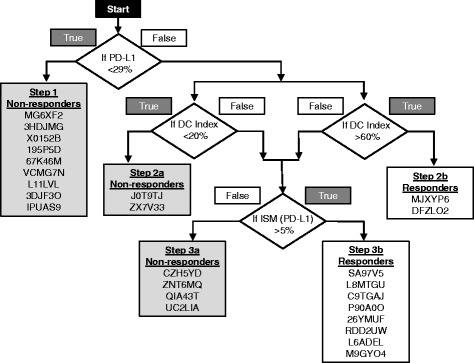
A decision tree was used to identify PD-1 drug responder status. At step 1, 9 patients with PD-L1 expression below 29.0% were identified as PD-1 drug non-responders. The remaining 16 patients (including patient SA97V5) with PD-L1 expression equal to or greater than 29.0% proceeded to step 2. At Steps 2a and 2b, 2 patients with dendritic cell infiltration index values below 20.0% were identified as non-responders and 2 patients with index values greater than 60.0% were identified as PD-1 drug responders. Twelve patients with index values greater than 20.0% (including patient SA97V5), but less than 60.0% proceeded to step 3. At Step 3, 4 patients with immunosuppressive molecule (ISM) values higher than that of their PD-L1 expression with a margin of greater than 5.0%, were identified as non-responders (Step 3a) and 8 patient-specific models with values lower than that of their PD-L1 expression with a margin of greater than 5.0% were identified as responders (Step 3b, including patient SA97V5). Mismatch patients GI7AGZ, 2FCOH7, F3FK2W were not listed

Differences among the expression of 14 molecules were used in a 3-step process to sort patients into those that would or would not respond to PD-1 immunotherapy (Fig. 2). Patients were sorted by their PD-L1 expression (Step 1), their dendritic cell infiltration index (Steps 2a and b), and their immunosuppressive molecule expression (Steps 3a and b).

### Signal pathways

Graphic representations of the simulation model networks for each patient-specific model and the underlying network relationships were created as previously described [24] to identify similarities in patient-specific signaling pathways and to identify the influence of the pathway intermediates altered by the patient deleterious gene mutation profiles.

### Simulation model validations

A series of internal control check analyses were used to validate the cancer network input and output data. These control checks monitored a) the effects of select pathway molecule over-expression or knockdown on pathway predictions, b) the effects of select drugs on pathway predictions, and c) the effects of activation, regulation, and cross-talk interactions among pathway intermediates on pathway predictions.

A cross-validation approach was used to assess the match scores of the PD-1 predicted responses against the PD-1 clinical responses in the Rizvi et al. 2015 Discovery dataset vs. the Validation dataset [14]. The datasets were then pooled and re-partitioned into two new Training and Test datasets. A similar cross-validation approach was then used to assess the match scores of the PD-1 predicted responses vs. the PD-1 clinical responses. Differences between the match rates of the PD-1 predicted responses and the PD-1 clinical responses were performed via chi-square test or Fisher’s exact as previously described [24]. All statistical tests utilized a 0.05 level of significance.

## Results

### Simulation models

In this retrospective study, we created 29 of 34 separate and patient-specific simulation models from the exome sequencing information for each of the 34 patients listed in Supplement Table 3 of the Rizvi et al study [15]. In the Discovery dataset, 13 of 16 patients had sufficient information to create simulation models and patients RYRJFL, IYXPLI, and GOFKQI did not (Additional file 2: Table S2). In the Validation dataset, 16 of 18 patients had sufficient information to create computational simulation models and patients 6NLFT5 and 32I5VC did not (Additional file 2: Table S2). The 5 patients with insufficient information were omitted from this study since their deleterious gene mutation profiles lacked driver genes and we were unable to achieve an increase in tumor phenotypes of proliferation and viability with the subset of gene aberrations reported. The remaining patients in the Discovery dataset (*n* = 13) contained 5 clinical responders and 8 clinical non-responders and the patients in the Validation dataset (*n* = 16) contained 6 clinical responders and 10 clinical non-responders (Table 1). Objective responses to PD-1/PD-L1 immunotherapies are known to vary. For example, objective responses to PD-1 immunotherapies for NSCLC were reported to range from 19.0–21.0% and objective responses to PD-L1 immunotherapies for NSCLC were reported to range from 10.0–23.0% [1, 2]. Hence the proportion of more non-responders than responders in this sample size was representative of the responses previously reported in larger patient populations.

### Patient molecule responses

Results reported for patient SA97V5 were used as an example to clearly illustrate the process of model creation, model prediction, and model validation.

Modeled PD-L1 expression ranged from − 8.3% (patient 67K46M) to 185.5% (patient M9GYO4) (Table 1). Patient SA97V5 had a PD-L1 expression value of 67.0%.

Modeled chemokine expression was used to create a dendritic cell infiltration index. This index was a weighted function of the percentage change of each of the 9 individual chemokines (Table 1) and ranged from 4.20 (patient 67K46M) to 79.85 (patient DFZLO2). Patient SA97V5 chemokine expression for CCL2 (28.7%), CCL3 (14.3%), CCL4 (27.2%), CCL5 (13.4%), CCL7 (38.0%), CCL11 (36.9%), CCL20 (30.6%), CX3CL (32.9%), and CXCL14 (− 3.3%); formula details; and calculations for creating the index value of 23.9% are shown in Additional file 6: Table S4.

Modeled expression profiles for 14 immunosuppressive molecules, including those for patient SA97V5, are also shown in Table 1.

### Clinical response projections

The expression of PD-L1, dendritic cell infiltration index, and immunosuppressive molecules were used in a 3-step process to sort patients into those that would or would not respond to PD-1 immunotherapy (Fig. 2).

**Fig. 3:**
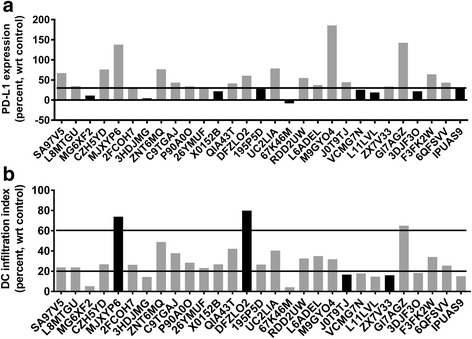
Patient-specific simulation models were used to predict the expression of PD-L1 (**a**) and at step 1 of the decision tree, 9 patients (black bars) with predicted PD-L1 expression below 29.0% (bold line) were identified as PD-1 drug non-responders. Patient SA97V5 had a predicted PD-L1 expression of 67.0%. Patient-specific simulation models were used to predict the expression of chemokines used to create a dendritic cell (DC) infiltration index (**b**). At step 2a of the decision tree, 2 patients (black bars) with index values greater that 60.0% (bold line) were identified as PD-1 drug responders and 2 patients (black bars) with index values less than 20.0% (black line) were identified as PD-1 drug non-responders. Patient SA97V5 had a predicted DC infiltration index of 23.9%

At step 1, 9 patients with PD-L1 expression below 29.0% were identified as PD-1 drug non-responders (Figs. 2 and 3a). The remaining 16 patients with PD-L1 expression equal to or greater than 29.0% proceeded to step 2. Patient SA97V5 had a PD-L1 expression value of 67.0% and proceeded to step 2.

At Step 2, 2 patients with dendritic cell infiltration index values below 20.0% were identified as non-responders (Figs. 2 and 3b) and 2 patients with index values greater than 60.0% were identified as PD-1 drug responders (Figs. 2 and 3b). One mismatch occurred at this step. Clinical non-responder GI7AGZ with a dendritic cell index of 64.9% was misidentified as a PD-1 responder (Fig. 3b). Twelve patients with index values greater than 20.0%, but less than 60.0% proceeded to step 3. Patient SA97V5 had a dendritic cell infiltration index of 23.9% and proceeded to step 3.

**Fig. 4:**
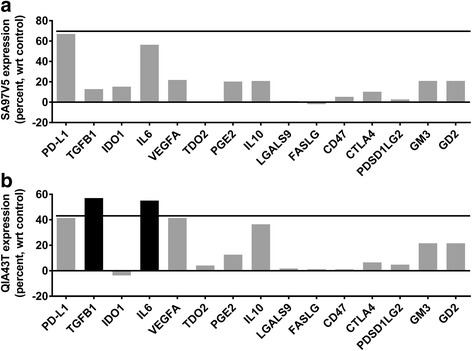
Patient-specific simulation models were used to predict the expression of 14 immunosuppressive molecules. At step 3 of the decision tree, patients with immunosuppressive molecule predictions higher than that of PD-L1 with a margin of greater than 5.0% (bold line), were considered to be non-responders and patient-specific models with predictions lower than that of PD-L1 with a margin of greater than 5.0% were considered to be responders. Eight remaining patients were identified as responders and 4 remaining patients were identified as non-responders. Patient SA97V5 (**a**) had all 14 molecules below the threshold and was identified as a PD-1 drug responder. Patient QIA43T (**b**) had 2 molecules above the threshold and was identified as a PD-1 drug non-responder

At Step 3, 4 patients with immunosuppressive molecule values higher than that of their PD-L1 expression with a margin of greater than 5.0% were considered to be non-responders (Fig. 2, Step 3a) and 8 patient-specific models with values lower than that of their PD-L1 expression with a margin of greater than 5.0% were considered to be responders (Fig. 2, Step 3b). Three mismatches occurred at this step. Clinical responder patient 2FCOH7 had an immunosuppressive molecule expression profile of a non-responder: vascular endothelial growth factor (VEGF), cytotoxic T-lymphocyte-associated protein 4 (CTLA4), ganglioside GM3 (GM3), and ganglioside GD2 (GD2) were all higher than that of PD-L1 with a margin of greater than 5.0%. Clinical non-responder patients F3FK2W and 6QFSVV had immunosuppressive molecule expression profiles of responders. Patient SA97V5 had all 14 immunosuppressive molecules below the threshold of PD-L1 and was identified as a PD-1 drug responder (Figs. 2 and 4a) and patient QIA43T had molecules TGFB1 and IL6 above the threshold of PD-L1 and was identified as a PD-1 drug non-responder (Figs. 2 and 4b).

Patient-specific model predictions in the Discovery and Validation datasets were also checked using Weka 3 [32] and SMO support vector machine with a normalized polynomial kernel had the best performance (Additional file 7: Table S5). The relationship between PD-L1 expression and predicted TGFB1 expression using Weka 3 algorithms for all patients in the dataset is shown in Additional file 8: Figure S2 and similar trends were seen when comparing the PD-L1 expression level to the other 13 predicted molecules. Weka 3 correctly identified 24 out of 29 patients whereas the computational simulation models correctly identified 25 of 29 patients.

### Model validation

In the cross-validation analysis of the match scores between the Rizvi et al. 2015 Discovery and Validation datasets, there were no significant differences between the match scores of non-responders and responders in the PD-1 clinical response group (38.5% vs. 37.5%; *p* = 0.9577, Additional file 9: Table S6) and PD-1 predicted response group (30.8% vs. 56.3%; *p* = 0.2642, Additional file 9: Table S6). Even though the Discovery dataset had a higher match score rate among the PD-1 clinical response group and the PD-1 predicted response group than the Validation dataset (92.3% vs. 81.2%, respectively), there was no significant difference between the two datasets (*p* = 0.6059).

Similarly, in the cross-validation analysis of the match scores between the Training and Test datasets, there were no significant differences between the match scores of non-responders and responders in the PD-1 clinical response group (38.9% vs. 36.4%; *p* = 0.9999, Additional file 9: Table S6) and PD-1 predicted response group (44.4% vs. 45.5%; p = 0.9577, Additional file 9: Table S6). In the Training dataset, the PD-1 predicted responses had an 83.3% match score with the PD-1 clinical responses and in the Test dataset the PD-1 predicted responses had a 90.9% match score with the PD-1 clinical responses. Again, there was no significant difference between the two datasets (p = 0.9999).

### Predicted pathway comparisons

**Fig. 5:**
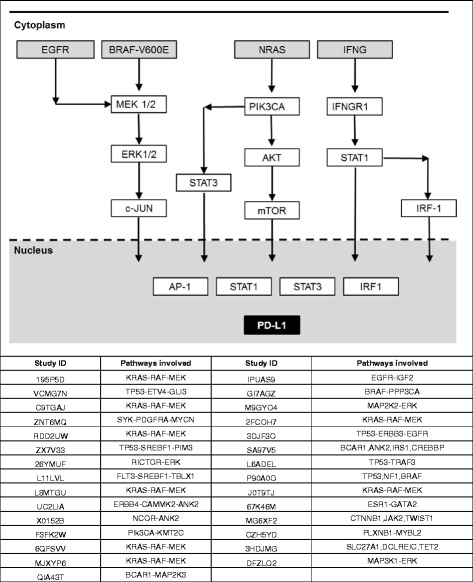
The expression of PD-L1 was influenced via a number of signaling pathways. Activating signals were processed via the ERK signaling pathway (via EGFR; B-Raf proto-oncogene, serine/threonine kinase, BRAF-V600E; mitogen-activated protein kinase kinase 1/2, MEK1/2; mitogen-activated protein kinase kinase 1, MAP2K1; MAP2K2; ERK1/2; mitogen-activated protein kinase 3, MAPK3; mitogen-activated protein kinase 1, MAPK1; and Jun proto-oncogene, c-Jun). Activating signals were processed via the EGFR signaling pathway (via neuroblastoma RAS viral oncogene homolog, NRAS; phosphatidylinositol-4,5-bisphosphate 3-kinase catalytic subunit alpha, PIK3CA; V-akt murine thymoma viral oncogene homolog, AKT; mechanistic target of rapamycin, MTOR; and STAT3). Also, activating signals were processed via the interferon gamma (IFNG) pathway (via IFNG; interferon gamma receptor 1, IFNGR1; signal transducer and activator of transcription 1, STAT1; and interferon regulatory factor 1, IRF1). Pathway signals converge to activation factors Activator protein 1 (AP1), STAT1, STAT3, and IRF1 leading to transcription of PD-L1 genes. Common pathways were utilized among a number patient-specific simulation models. Patient C9TGAJ (KRAS mutation), patient RDD2UW (KRAS mutation), patient M9GYO4 (MAP2K2 mutation), and patient DFZLO2 (MAP3K1 mutation) involved the ERK activation pathway. Patient P90A0O (BRAF1, TP53 mutations) and patient L8MTGU (KRAS, TP53 mutations) involved the ERK activation and apoptotic pathways

Deleterious gene mutations in patients were mapped to unique and common signaling pathways involved in PD-L1 expression (Fig. 5). Common pathways were utilized among a number of patient-specific models. Mutations in patient C9TGAJ (kirsten rat sarcoma viral oncogene homolog, KRAS mutation), patient RDD2UW (KRAS mutation), patient M9GYO4 (mitogen-activated protein kinase kinase 2, MAP2K2 mutation), and patient DFZLO2 (mitogen-activated protein kinase kinase kinase 1, MAP3K1 mutation) altered the extracellular signal-regulated kinase (ERK) activation pathway. Mutations in patient P90A0O (B-Raf proto-oncogene 1, BRAF1 mutation and tumor protein p53, TP53 mutation) and patient L8MTGU (KRAS mutation) altered ERK activation and apoptotic pathways. Mutations in patient SA97V5 (breast cancer anti-estrogen resistance protein 1, BCAR1 mutation; ankyrin-2, ANK2 mutation; insulin receptor substrate 1, IRS1 mutation; and cAMP response element-binding binding protein, CREBBP mutation), patient 26YMUF (rapamycin-insensitive companion of TOR, RICTOR mutation and ERK mutation), and patient L6ADEL (TP53 mutation and TNF receptor-associated factor, TRAF3 mutation) all had non-KRAS and non-B-Raf proto-oncogene (BRAF) driven activation pathways of PD-1 drug responder status.

Models also reinforced the association between PD-L1 expression and the presence of a KRAS mutation (or high ERK activation). Thus if a patient responder was found to have a KRAS mutation or positive regulator around mitogen-activated protein kinase kinase (MEK) pathway, this may identify a means to regulate PD-L1 by the MEK mediated pathway. A KRAS/BRAF/MEK related mutation in the profile leads to stronger expression of PD-L1 in the profile. Patients 195P5D, J0T9TJ, and 6QFSVV had KRAS mutations but were non-responders to PD-L1 inhibitor indicating complex and additional factors and pathways driving PD-L1 expression and response to the checkpoint inhibitor.

### KRAS mutations and PD-L1 expression

To further affirm the association between the presence of KRAS mutations or KRAS co-mutations and PD-L1 expression, 2 additional datasets [33, 34] were modelled beyond the Supplement Table 3 of the Rizvi et al study [15].

KRAS mutations in lung adenocarcinoma were reported to be associated with co-mutations in TP53. In modeled simulations of the Dong, et al. dataset [33], the KRAS+TP53 co-mutation (KP subgroup) was predicted to increase PD-L1 expression. The KRAS+TP53 co-mutation had higher levels of predicted PD-L1 expression than the KRAS mutation and TP53 mutation alone.

KRAS mutations in lung adenocarcinoma were also reported to be associated with co-mutations in STK11/LKB1 (the KL subgroup) [34]. In modeled simulations of the Skoulidis, et al. dataset [34], KRAS+STK11+KEAP1 co-mutation was predicted to reduce PD-L1 expression. The KRAS+STK11+KEAP1 co-mutation had lower levels of predicted PD-L1 expression than the KRAS+TP53 co-mutation.

KRAS mutations in lung adenocarcinoma were reported to be associated with co-mutations in TP53 (KP subgroup) and CDKN2A/B [34]. In modeled simulations of the Skoulidis, et al. dataset [34], KRAS+CDKN2A/B co-mutations (KC subgroup) were predicted to reduce PD-L1 expression. KRAS+CDKN2A/B co-mutation had lower levels of predicted PD-L1 expression than the KRAS+STK11+KEAP1 co-mutation and the KRAS+TP53 co-mutation.

## Discussion

In this retrospective study, we used a recent dataset from NSCLC patients treated with pembrolizumab and identified deleterious gene mutational profiles in patient exomes. We annotated the deleterious gene mutational profiles into a cancer network to create NSCLC patient-specific predictive computational simulation models. We used these models as a tool to identify and validate a profile of 24 chemokines and immunosuppressive molecules that could accurately affirm expression of PD-L1 and predict patient clinical responses to PD-1 immunotherapy. We found that patient tumor cell genomics influenced cell signaling and altered the expression of PD-L1, 9 chemokines, and 14 immunosuppressive molecules. We also found that expression profiles of these 24 chemokines and immunosuppressive molecules could be used to identify patients who would or would not respond to PD-1 immunotherapy. Adding chemokine and immunosuppressive molecule expression profiles to a predicted PD-L1 profile allowed models to achieve a greater than 85.0% predictive correlation among predicted and reported patient clinical responses. This differentiated patients who would and would not benefit from PD-1 or PD-L1 immunotherapies. To validate our results, we used retrospective correlation of our simulation models against patient genomic signatures and clinical outcome data that was available in the NSCLC cohort of the Rizvi et al. study [15]. We also used Weka 3 to validate predictions determined using the predictive computational simulation models. The Weka 3 results were similar to that generated via machine-learning methods and the Chi-square test was used to show no differences among the match rate results in these datasets. It is important to note that expanding PD-L1 expression profiles to include 23 additional chemokine and immunosuppressive molecule expression responses allowed models to achieve a greater than 85.0% correlation among predicted and reported patient clinical responses.

The 24 molecules used in this study have immunosuppressive properties. The role of PD-L1 in tumor pathogenesis is well known. Increased expression of PD-L1 on tumor cells inhibits T-cell proliferation, reduces T-cell survival, inhibits cytokine release, and promotes T-cell apoptosis [3, 35–38]. This leads to T-cell exhaustion and adaptive tumor immunosuppression [39].

Cytokines also have a role. Cytokine scores were recently found to be associated with overall survival in CheckMate 017 and 057 (both for nivolumab and docetaxel treated patients) [40]. In this present study, 9 chemokines were selected that chemoattractant dendritic cells [41–43]. In other studies, dendritic cells were present in NSCLC tumors [42, 44] and dendritic cell infiltration was reported to be an independent prognostic factor for NSCLC [44]. The simulation models here captured the role of dendritic cells by predicting chemokine expression in the form of a functional dendritic cell index (Additional file 6: Table S4).

Fourteen molecules were included that have pleiotropic functions including immunosuppressive properties (Additional file 1: Table S1). IL6, can prevent dendritic cell maturation, prime tumor-specific T-cells via signal transducer and activator of transcription 3 (STAT3) signaling, inhibit NF-κB binding activity, and inhibit C-C chemokine receptor type 7 (CCR7) expression [45–47]. IL10 can impair dendritic cell function and protect tumor cells from cytotoxic T-cell-mediated cytotoxicity by downregulating transporter-associated with antigen processing (TAP)1 and TAP2 [48, 49]. TGFβ can alter immune surveillance of regulatory T-cells. It represses CTL-mediated tumor cytotoxicity by altering the expression of perforin, granzyme A, granzyme B, Fas ligand (FASL), and IFNγ [49–51]. VEGF is a marker of tumor invasion and metastasis and can inhibit maturation of dendritic cells [44, 52]. IDO, a tryptophan-metabolizing enzyme that limits tryptophan, inhibits the proliferation of lymphocytes, and contributes to peripheral immunologic tolerance [53–56]. Increased IDO production by cancer cells down regulates natural killer (NK) receptors and induces NK cell apoptosis. It induces cell cycle arrest, decreases activation, and increases apoptosis in cytotoxic T-cells. Tryptophan 2, 3-dioxygenase 2 (TDO2) inhibits tryptophan 2,3-dioxygenase [57]. Prostaglandin E2 (PGE2) suppresses NK cell function through the E_2_ prostaglandin receptor 4 (EP4) [58]. Lectin, galactoside-binding, soluble, 9 (LGALS9) mediates T-cell dysfunction and T-cell senescence [59]. Cluster of Differentiation 47 (CD47) is a negative regulator of dendritic cells binding to signal regulatory protein (SIRP) on dendritic cells and directly repressing dendritic cell phagocytosis, maturation, and production of IFNγ [47]. CTLA4 restrains the adaptive immune response of T-cells towards tumor-associated antigens [60–62]. Gangliosides GM3 and GD2 induce monocyte apoptosis and impair differentiation to dendritic cells [63].

Using a 24 molecule expression profile allowed computational models to achieve a greater than 85.0% predictive correlation. However, this list was not exclusive and incorporating additional molecules into patient NSCLC-specific expression profiles may have merit and improve computational model accuracy. These included Lymphocyte activation gene-3 (LAG-3), T cell immunoglobulin-3 (TIM-3), and T cell immunoglobulin and ITIM domain (TIGIT) co-inhibitory receptors [64]. LAG-3 is a co-inhibitory receptor upregulated on activated CD4+ T cells, CD8+ T cells, and subsets of natural killer (NK) cells [64–66]. It impairs T cell proliferation and cytokine production and alters NK cell cytotoxicity and cytokine production. TIM-3 is a cell surface molecule expressed on IFNγ-producing CD4+ T helper 1 cells, CD8+ T cytotoxic 1 T cells, NK cells, monocytes, and dendritic cells [64]. TIM-3 dampens the development of protective immunity and TIM-3 blockade improves cell function. In patients with NSCLC, co-blockade of the TIM-3 and PD-1 pathways suppresses tumor growth. TIGIT is another co-inhibitory receptor expressed on NK cells, T cells, and Treg cells [64]. CD155, CD112, and TIGIT ligands suppress immune responses through CD155 on dendritic cells. TIGIT is thought to work with PD-1 and TIM-3 to attenuate T cell responses and promote T cell dysfunction.

After the NSCLC patient-specific predictive computational simulation models were created and the profiles of 24 chemokines and immunosuppressive molecules were predicted, we created a decision tree to identify patients who would or would not respond to PD-1 immunotherapy. Decision cutoffs were established at 29.0% PD-L1 expression (Step 1), < 20.0% dendritic cell infiltration (Step 2a), > 60.0% dendritic cell infiltration (Step 2b), and immunosuppressive molecule expression as < PD-L1 with a margin of greater than 5.0% (Step 3) (Fig. 2). The decision tree was robust and had built-in redundancy. Basing the PD-L1 drug responder status on 3 separate predicted criteria allowed a responder/non-responder not identified at one step to be identified at a later step. Also the thresholds were specific. At 29.0% PD-L1 expression (Step 1), 9 non-responder patients were identified. Decreasing the PD-L1 expression cutoff from 29.0% to 25.0% identified only 6 non-responders. Increasing the PD-L1 expression cutoff from 29.0% to 35.0% identified a number of false negatives and setting the PD-L1 expression cutoff at 35.0% identified up to 13 non-responder patients: the three additional patient responders L8MTGU, P90A0O, and 26YMUF would be falsely identified as non-responders.

A diversity of signaling pathways are reported to be involved in the expression and regulation of PD-L1 [67–69] and these pathways were observed in expression and regulation of PD-L1 in this study (Fig. 5). Responder patients had mutations around the rapidly accelerated fibrosarcoma (RAF)-rat sarcoma (RAS)-ERK pathway including KRAS/BRAF and MEK-related mutations that predicted the profiles to have stronger expression of PD-L1. However, the presence of KRAS cannot be the only criteria for predicting strong expression of PD-L1 and thus a likely PD-1 drug responder, since there were non-responder profiles that also had KRAS mutations. We observed that matched predicted and clinical non-responder patients 195P5D and J0T9TJ and mismatched predicted responder and clinical non-responder patient 6QFSVV all had KRAS mutations (Additional file 2: Table S2, Fig. 5).

Recent studies support the concept that NSCLC is not a homogeneous disease and at least 3 subtypes of KRAS mutations involving LKB1 or TP53 can be identified. The tumors with these mutations have different PD-L1 expression patterns (higher in KRAS mutations and TP53 mutations) and different sensitivities to immune checkpoint blockade. Thus the effects of KRAS mutations and KRAS co-mutations on PD-L1 expression was further assessed using 2 additional datasets [33, 34] beyond the Supplement Table 3 of the Rizvi et al study [15].

Dong, et al. [33] reported that TP53 and KRAS mutations may predict which patients would or would not respond to PD-1 immunotherapy. Modeling the dataset in their study, we predicted that KRAS+TP53 co-mutation (KP Subgroup) would lead to increased PD-L1 expression. Skoulidis, et al. [34] reported that KRAS mutations in lung adenocarcinoma were associated with co-mutations in STK11/LKB1 (the KL subgroup) [34]. KL tumors had high rates of KEAP1 mutations with lower PD-L1 expression. Modeling the dataset in their study, we predicted that KRAS+STK11+KEAP1 co-mutations (KL Subgroup) also would lead to reduced PD-L1 expression. We predicted that KRAS+CDKN2A/B co-mutation (KC Subgroup) would lead to reduced PD-L1 expression. There was a reduction in positive regulation due to reduction in AMPK, mTOR pathway and also due to KEAP1 loss of function. There was an increase in the WT TP53 mediated inhibitory regulation of PD-L1 expression. This was a novel finding based on network analysis.

Furthermore, PTEN deletion [70], PI3K mutations [71] and MYC overexpression [72] have also been recently characterized as oncogenic mechanisms leading to PD-L1 expression.

The techniques described in this retrospective study have application. Although the techniques were complicated and need more extensive validation with larger datasets, their utility in clinical practice is possible. Profiling of tumors is becoming more main stream for precision personalized medicine. The approach may not necessarily be expensive, but in fact provides more utility to the generated profiling data for most tumor samples.

## Conclusions

Patient tumor cell genomics were found to influence cell signaling with downstream effects on the expression of 24 chemokines and immunosuppressive molecules. This allowed us to establish patient-specific profiles of these molecules that could be used to predict patient clinical responses with greater than 85.0% correlation among predicted and reported patient clinical responses. Developing a workflow incorporating immunosuppressive molecules could a) be used as a potential complementary assay to affirm IHC results or used as an alternate assay where IHC in unfeasible, b) affirm patient PD-1 and PD-L1 drug responder status, c) as a method to determine influencing factors on PD-L1 expression, and d) as a potential clinical decision support system facilitating selection of therapies based on individual patient mutational profiles. The latter application used shortly after cancer diagnosis and just before cancer treatment could generate important patient-specific treatment options that could assist clinicians in selecting appropriate mono-therapies or combination therapies.

## Additional files

Additional file 1: **Table S1.** Molecules with immunosuppressive functions used in simulation models to predict PD-1 drug responder status. (DOCX 55 kb)

Additional file 2: **Table S2.** Individual mutational profiles of patient drug responders (*n* = 11) and nonresponders (*n* = 18). (DOCX 19 kb)

Additional file 3: **Figure S1.** A schematic pyramid showing the levels of information used to develop the validated, cancer network. This network was created from published reports on cell receptors, signaling pathways, pathway signaling intermediates, activation factors, transcription factors, and enzyme kinetics. Information on each pathway node, its functionality, and its links with other genes, proteins, and pathways was manually researched, analyzed, curated, and aggregated to construct the integrated network maze. Every process or reaction was modeled mathematically using Michaelis Menton kinetics, mass action kinetics, and variations of these representations using ordinary differential equations (ODEs). Modeled events included but were not limited to interactions at the cell surface (e.g., binding of ligands to receptors, etc.), metabolic and cell signaling (e.g., signal pathway events, cross talk interactions among pathways, feedback control, etc.), activation and regulation of genes (e.g., activation links of transcription factors, etc.), intracellular processes such as proteasomal degradation, endoplasmic reticulum (ER) stress, oxidative stress, DNA damage and repair pathways, and cell cycle pathways. Time-dependent changes in signaling pathway fluxes of every biological reaction modeled utilizing modified ODEs were solved with a proprietary solver. Models were validated with a series of internal control analysis checks on predictions. These checks included assessing the effects of pathway molecule over-expression or knockdown on pathway predictions; effects of drugs on pathway predictions; and activation, regulation, and cross-talk interactions among pathway intermediates on pathway predictions. (DOCX 53 kb)

Additional file 4: Supplementary Materials and Methods. (DOCX 109 kb)

Additional file 5: **Table S3.** An example of the predictive computational modeling process. Specific details on an annexure section of the PD-L1 pathway show the step-by-step reactions, mechanisms, and reaction equations that occur. Such reactions also occurred in all of the other pathways. (DOCX 102 kb)

Additional file 6: **Table S4.** Creation of the dendritic cell infiltration index for the patient SA97V5-specific simulation model. Chemokines CCL11, CCL20, CCL2, CCL3, CCL4, CCL5, CCL7, CX3CL1, and CXCL14, capable of trafficking of dendritic cells into the tumor microenvironment, were used to create the index. Individual chemokine percent expression (with respect to non-tumorigenic baseline controls) was predicted and given weightage so as to normalize the total to 1. The index was then calculated to be the sum of each prediction % change * weightage. (DOCX 16 kb)

Additional file 7: **Table S5.** Analysis of the Discovery and Validation datasets was performed using Weka 3. The first number in each column represented the number of patient treatment responses correctly classified by the model. The second number represented the number of incorrectly classified patient treatment responses. The GOAL row at the bottom of each column described the number of correctly and incorrectly classified patients in the simulation models. The Test Set columns described the output from applying the model trained on the Discovery set to the Validation set. The “Test and Train” columns described test set accuracy (test set column) plus the training error (results obtained by applying the model to the training set, i.e. training error). (DOCX 19 kb)

Additional file 8: **Figure S2.** An example of the relationship between PD-L1 expression and predicted TGFB1 expression using Weka 3 algorithms for all patients in the dataset. Similar trends were seen when comparing the PD-L1 expression level to the other 13 predicted molecules. For this, the number of gene mutations identified for each patient ranged from 2 to 36 with a total of 264 unique genes between all patients. This categorical data was preprocessed and expanded into a gene vector of length 264 to represent each of the unique genes. For each gene in the vector, the data was represented in binary; a 1 was assigned if the patient had a mutation in this gene, a 0 otherwise. Two datasets, one including gene mutations (Molecules and Gene Mutations) and one without (Molecules), were both used to learn prediction models. The Discovery and Validation datasets were determined based on the split provided to allow for comparable results. The performance of a subset of these models on the testing and training sets for both Molecules and Molecules and Gene Mutations datasets are shown. The SMO support vector machine with a normalized polynomial kernel had the best performance when applied to the molecule dataset. This model correctly identified 24 out of 29 patients whereas the simulation models correctly identified 25 of 29. This was only a difference of one match between the two prediction methods. Still, several other methods, while not performing as well overall, were able to identify 9 patients in the test dataset accurately. This was near the computational simulation model prediction capability in which 10 patients were successfully identified in the test dataset. In general, adding the gene mutation data to the molecule data either maintained or decreased the performance of a model. (DOCX 4114 kb)

Additional file 9: **Table S6.** Comparisons of clinical and predicated responses and match scores. We used a cross-validation approach to assess the match scores in Table 1 of the PD-1 predicted responses against the PD-1 clinical responses in the Rizvi et al. 2015 Discovery dataset vs. the Validation dataset. We then pooled and re-partitioned the dataset into two new Training and Test datasets. We then used a similar cross-validation approach to assess the match scores of the PD-1 predicted responses vs. the PD-1 clinical responses. (DOCX 17 kb)

## Abbreviations

AKT: V-akt murine thymoma viral oncogene homolog
ANK2: Ankyrin-2
AP1: Activator protein 1
BCAR1: Breast cancer anti-estrogen resistance protein 1
BRAF: B-Raf proto-oncogene
BRAF1: B-Raf proto-oncogene 1
BRAF-V600E: B-Raf proto-oncogene, serine/threonine kinase
CCR7: C-C chemokine receptor type 7
CD47: Cluster of Differentiation 47
CDKN2A/B: Cyclin-dependent kinase Inhibitor 2A
c-Jun: Jun proto-oncogene
CREBBP: cAMP response element-binding binding protein
CTLA4: Cytotoxic T-lymphocyte-associated protein 4
EP4: E_2_ prostaglandin receptor 4
ERK: Extracellular signal-regulated kinase
FASL: Fas ligand
GD2: Ganglioside GD2
GM3: Ganglioside GM3
IDO: Indoleamine-pyrrole 2,3-dioxygenase
IFNG: Interferon gamma
IFNGR1: Interferon gamma receptor 1
IRF1: Interferon regulatory factor 1
IRS1: Insulin receptor substrate 1
KEAP1: Kelch-like ECH-associated protein 1
KRAS: Kirsten rat sarcoma viral oncogene homolog
LAG3: Lymphocyte-activation protein 3
LGALS9: Lectin, galactoside-binding, soluble, 9
LKB1: Liver kinase B1
MAP K1: Mitogen-activated protein kinase 1
MAP K3: Mitogen-activated protein kinase 3
MAP2K1: Mitogen-activated protein kinase kinase 1
MAP2K2: Mitogen-activated protein kinase kinase 2
MAP3K1: Mitogen-activated protein kinase kinase kinase 1
MEK: Mitogen-activated protein kinase kinase
MEK1/2: Mitogen-activated protein kinase kinase 1/2
MTOR: Mechanistic target of rapamycin
MYC: MYC proto-oncogene, bHLH transcription factor
NK: Natural killer
NKX2–1: NK2 Homeobox 1
NRAS: Neuroblastoma RAS viral oncogene homolog
NSCLC: Non-small cell lung cancer(s)
ODE: Ordinary differential equation(s)
PGE2: Prostaglandin E2
PI3K: Phosphatidylinositol-4,5-bisphosphate 3-kinase
PIK3CA: Phosphatidylinositol-4,5-bisphosphate 3-kinase catalytic subunit alpha
PTEN: Phosphatase and tensin homolog
RAF: Rapidly accelerated fibrosarcoma
RAS: Rat sarcoma
RICTOR: Rapamycin-insensitive companion of TOR
SIRP: Signal-regulatory protein
STAT1: Signal transducer and activator of transcription 1
STAT3: Signal transducer and activator of transcription 3
STK11: Serine/threonine kinase 11
TAP: Transporter-associated with antigen processing
TDO2: Tryptophan 2, 3-dioxygenase 2
TIGIT: T-Cell Immunoreceptor With Ig And ITIM Domains
TIM-3: T-cell immunoglobulin and mucin-domain containing-3
TP53: Tumor protein p53
TRAF3: TNF receptor-associated factor
VEGF: Vascular endothelial growth factor
