# Correction to: Genomics of NSCLC patients both affirm PD-L1 expression and predict their clinical responses to anti-PD-1 immunotherapy

**DOI:** 10.1186/s12885-018-4200-5

**Published:** 2018-04-12

**Authors:** Kim A. Brogden, Deepak Parashar, Andrea R. Hallier, Terry Braun, Fang Qian, Naiyer A. Rizvi, Aaron D. Bossler, Mohammed M. Milhem, Timothy A. Chan, Taher Abbasi, Shireen Vali

**Affiliations:** 1 Iowa Institute for Oral Health Research, College of Dentistry, The University of Iowa, 801 Newton Road, Iowa City, IA 52242 USA; 2 Cellworks Research India Ltd., Whitefield, Bangalore, 560066 India; 3 Biomedical Engineering, The University of Iowa, 5318 SC, Iowa City, IA 52242 USA; 4 Division of Biostatistics and Research Design, College of Dentistry, The University of Iowa, 801 Newton Road, Iowa City, IA 52242 USA; 5 Division of Hematology/Oncology, Columbia University Medical Center, 177 Fort Washington Avenue, New York, NY 10032 USA; 6 Molecular Pathology Laboratory, Department of Pathology, University of Iowa Hospitals and Clinics, 200 Hawkins Dr., C606GH, Iowa City, IA 52242 USA; 7 Clinical Services, Experimental Therapeutics, Melanoma and Sarcoma Program, Holden Comprehensive Cancer Center, The University of Iowa, Iowa City, IA 52242 USA; 8 Department of Radiation Oncology, Human Oncology and Pathogenesis Program, Immunogenomics and Precision Oncology Platform, Memorial Sloan Kettering Cancer Center, New York, NY 10065 USA; 9 Cellworks Group, Inc., 2033 Gateway Place Suite 500, San Jose, CA 95110 USA

## Correction

It has been highlighted that in the original manuscript [1] Table S3 ‘An example of the predictive computational modeling process. Specific details on an annexure section of the PD-L1 pathway show the step-by-step reactions, mechanisms, and reaction equations that occur. Such reactions also occurred in all of the other pathways’ was omitted and did not appear in the Additional files. The original article has been updated.

## References

[1] Brogden KA (2018). Genomics of NSCLC patients both affirm PD-L1 expression and predict their clinical responses to anti-PD-1 immunotherapy. BMC Cancer.

