# Supplementary material for: Genomics of NSCLC patients both affirm PD-L1 expression and predict their clinical responses to anti-PD-1 immunotherapy
Source: BMC Cancer. 2018 Feb 27;18:225. doi: 10.1186/s12885-018-4134-y (PMC5897943; doi:10.1186/s12885-018-4134-y)
Supplement: Supplementary file 1 — Table S1. Molecules with immunosuppressive functions used in simulation models to predict PD-1 drug responder status. (DOCX 55 kb) [file 12885_2018_4134_MOESM1_ESM.docx]

| **Biomarker** | **Immunosuppressive function** | **References** |
| --- | --- | --- |
| CD47 | - Represses dendritic cell phagocytosis, maturation, and production of INFγ | [[1](#_ENREF_1)] |
| CTLA4 | - Restrains the adaptive immune response of T-cells towards tumor-associated antigens | [[2-4](#_ENREF_2)] |
| FASLG | - Tumor cells can overexpress FASLG. It triggers Fas-mediated apoptosis and induces apoptosis in T-cells expressing Fas, allowing for tumor progression | [[5](#_ENREF_5)] |
| GD2 | - Impairs dendritic cell differentiation from monocytes and induce their apoptosis | [[6](#_ENREF_6)] |
| GM3 | - Impairs dendritic cell differentiation from monocytes and induce their apoptosis | [[6](#_ENREF_6)] |
| IDO | - Inhibits the proliferation of lymphocytes - Acts on NK cells to down regulate receptors and induces NK cell apoptosis - Acts on cytotoxic T-cells to induce cell cycle arrest, decrease activation, and apoptosis | [[7-10](#_ENREF_7)] |
| IL10 | - Impairs dendritic cell function and protects tumor cells from cytotoxic T-cell-mediated cytotoxicity by downregulating transporter-associated with antigen processing (TAP)1 and TAP2 | [[11](#_ENREF_11), [12](#_ENREF_12)] |
| IL6 | - Prevents dendritic cell maturation, primes tumor-specific T-cells via STAT3 signaling, inhibits NF-κB binding activity, and inhibits CCR7 expression | [[1](#_ENREF_1), [13](#_ENREF_13), [14](#_ENREF_14)] |
| LGALS9 | - Mediates T-cell dysfunction and T-cell senescence | [[15](#_ENREF_15)] |
| PDCDILG2 | - Also known as PD-L2. It dramatically inhibits T cell receptor (TCR)-mediated proliferation and cytokine production by CD4^+^ T cells | [[16](#_ENREF_16)] |
| PGE2 | - Suppresses NK cell function primarily through the PGE2 receptor EP4 | [[17](#_ENREF_17)] |
| TDO2 | - Contributes to tumor immune resistance by inhibiting tryptophan 2,3-dioxygenase | [[18](#_ENREF_18)] |
| TGFβ | - Acts directly and through the generation of regulatory T-cells to alter immune surveillance - Represses the expression of cytolytic gene products perforin, granzyme A, granzyme B, Fas ligand (FasL), and IFNγ, which are responsible for CTL-mediated tumor cytotoxicity | [[12](#_ENREF_12), [19](#_ENREF_19), [20](#_ENREF_20)] |
| VEGF | - Inhibits maturation of dendritic cells | [[21](#_ENREF_21), [22](#_ENREF_22)] |

1. Ishii H, Tanaka S, Masuyama K: Therapeutic strategy for cancer immunotherapy in head and neck cancer. Adv Cell Mol Otolaryngol. 2015;3:27690.

2. Avogadri F, Yuan J, Yang A, Schaer D, Wolchok JD: Modulation of CTLA-4 and GITR for cancer immunotherapy. Curr Top Microbiol Immunol. 2011;344:211-44.

3. Mocellin S, Nitti D: CTLA-4 blockade and the renaissance of cancer immunotherapy. Biochim Biophys Acta. 2013;1836(2):187-96.

4. Grosso JF, Jure-Kunkel MN: CTLA-4 blockade in tumor models: an overview of preclinical and translational research. Cancer immunity. 2013;13:5.

5. Kim B, Lee HJ, Choi HY, Shin Y, Nam S, Seo G, Son DS, Jo J, Kim J, Lee J et al: Clinical validity of the lung cancer biomarkers identified by bioinformatics analysis of public expression data. Cancer Res. 2007;67(15):7431-8.

6. Peguet-Navarro J, Sportouch M, Popa I, Berthier O, Schmitt D, Portoukalian J: Gangliosides from human melanoma tumors impair dendritic cell differentiation from monocytes and induce their apoptosis. J Immunol. 2003;170(7):3488-94.

7. Munn DH, Zhou M, Attwood JT, Bondarev I, Conway SJ, Marshall B, Brown C, Mellor AL: Prevention of allogeneic fetal rejection by tryptophan catabolism. Science. 1998;281(5380):1191-3.

8. Lob S, Konigsrainer A, Rammensee HG, Opelz G, Terness P: Inhibitors of indoleamine-2,3-dioxygenase for cancer therapy: can we see the wood for the trees? Nature reviews Cancer. 2009;9(6):445-52.

9. Spranger S, Spaapen RM, Zha Y, Williams J, Meng Y, Ha TT, Gajewski TF: Up-regulation of PD-L1, IDO, and T(regs) in the melanoma tumor microenvironment is driven by CD8(+) T cells. Science translational medicine. 2013;5(200):200ra116.

10. Iversen TZ, Andersen MH, Svane IM: The targeting of indoleamine 2,3 dioxygenase -mediated immune escape in cancer. Basic Clin Pharmacol Toxicol. 2015;116(1):19-24.

11. Pinzon-Charry A, Maxwell T, Lopez JA: Dendritic cell dysfunction in cancer: a mechanism for immunosuppression. Immunol Cell Biol. 2005;83(5):451-61.

12. Rabinovich GA, Gabrilovich D, Sotomayor EM: Immunosuppressive strategies that are mediated by tumor cells. Annu Rev Immunol. 2007;25:267-96.

13. Menetrier-Caux C, Montmain G, Dieu MC, Bain C, Favrot MC, Caux C, Blay JY: Inhibition of the differentiation of dendritic cells from CD34(+) progenitors by tumor cells: role of interleukin-6 and macrophage colony-stimulating factor. Blood. 1998;92(12):4778-91.

14. Hegde S, Pahne J, Smola-Hess S: Novel immunosuppressive properties of interleukin-6 in dendritic cells: inhibition of NF-kappaB binding activity and CCR7 expression. FASEB J. 2004;18(12):1439-41.

15. Li H, Wu K, Tao K, Chen L, Zheng Q, Lu X, Liu J, Shi L, Liu C, Wang G et al: Tim-3/galectin-9 signaling pathway mediates T-cell dysfunction and predicts poor prognosis in patients with hepatitis B virus-associated hepatocellular carcinoma. Hepatology. 2012;56(4):1342-51.

16. Latchman Y, Wood CR, Chernova T, Chaudhary D, Borde M, Chernova I, Iwai Y, Long AJ, Brown JA, Nunes R et al: PD-L2 is a second ligand for PD-1 and inhibits T cell activation. Nat Immunol. 2001;2(3):261-8.

17. Holt D, Ma X, Kundu N, Fulton A: Prostaglandin E(2) (PGE (2)) suppresses natural killer cell function primarily through the PGE(2) receptor EP4. Cancer Immunol Immunother. 2011;60(11):1577-86.

18. Pilotte L, Larrieu P, Stroobant V, Colau D, Dolusic E, Frederick R, De Plaen E, Uyttenhove C, Wouters J, Masereel B et al: Reversal of tumoral immune resistance by inhibition of tryptophan 2,3-dioxygenase. Proc Natl Acad Sci U S A. 2012;109(7):2497-502.

19. Sauter ER, Nesbit M, Watson JC, Klein-Szanto A, Litwin S, Herlyn M: Vascular endothelial growth factor is a marker of tumor invasion and metastasis in squamous cell carcinomas of the head and neck. Clin Cancer Res. 1999;5(4):775-82.

20. Moutsopoulos NM, Wen J, Wahl SM: TGF-beta and tumors--an ill-fated alliance. Curr Opin Immunol. 2008;20(2):234-40.

21. Gabrilovich DI, Chen HL, Girgis KR, Cunningham HT, Meny GM, Nadaf S, Kavanaugh D, Carbone DP: Production of vascular endothelial growth factor by human tumors inhibits the functional maturation of dendritic cells. Nat Med. 1996;2(10):1096-103.

22. Inoshima N, Nakanishi Y, Minami T, Izumi M, Takayama K, Yoshino I, Hara N: The influence of dendritic cell infiltration and vascular endothelial growth factor expression on the prognosis of non-small cell lung cancer. Clin Cancer Res. 2002;8(11):3480-6.
