# Supplementary material for: Genomics of NSCLC patients both affirm PD-L1 expression and predict their clinical responses to anti-PD-1 immunotherapy
Source: BMC Cancer. 2018 Feb 27;18:225. doi: 10.1186/s12885-018-4134-y (PMC5897943; doi:10.1186/s12885-018-4134-y)
Supplement: Supplementary file 2 — Table S2. Individual mutational profiles of patient drug responders (n = 11) and nonresponders (n = 18). (DOCX 19 kb) [file 12885_2018_4134_MOESM2_ESM.docx]

| **Study ID** | **KRAS mutation present** | **Mutation component comparison** | |
| --- | --- | --- | --- |
| **Responders (n=11)** | | | |
| SA97V5 |  | BCAR1, UMPS, PPP1CC, NR1H2, ZEB2, ITGAM, KDM6B, TWIST1, NCOR2, CHN2, SMARCA4, KDM5C, RPIA, HK1, ATG13, BCL2L11, PTGER3, DOT1L, ULK3, PAK7, KMT2C, CREBBP, BCR, IRS1, TLN1, DUOX2, FIGF, AMPD1, PTPN11, PDIA3, ERBB3, CARD11, HDAC6, PREX1, TET1, ANK2 | |
| L8MTGU | **G12D** | CASP9, ZEB1, MYCN, DGKZ, TP53, CAPN2, BRCA1, CLTC, SIN3A, TCL1A, BCAR1, ATP2A3, FDPS, KRAS, GLI2, PRKDC, NOTCH1, NRG3, HGF, FGFR2, CDKN2A, KEAP1, SFRP1, ZEB2, FN1, SMO | |
| MJXYP6 | **G12C** | CCNG1, TYK2, RBL2, PRR5, KEAP1, DGKZ, APC, SALL3, DOCK4, AR, FANCD2, KRAS, SMARCA4, YY1AP1, CHD4, LCK, DUSP9, PPFIA3, SLC28A1, ADCY1, ADRM1, EPHB4, HIC1, MYCN, MED12, FLT4CARD11, SLC29A1 | |
| 2FCOH7 | **G12V** | CUX1, PTPRD, PTPRO, ALOX5, DRD2, NTRK2, TNIK, DGKA, MYBL2, RUNX3, ITGAD, PTPRK, KRAS, RGS6, GMPS, BCORL1, KMT2C, MED12, TP53, AR, RICTOR, CDX2, FGFR4, SMO, DUSP9, DOK2, EPHA7, CD40LG | |
| C9TGAJ | **G12C** | BCAR1, TP53, DYRK1B, MTA1, PARD3, CCR3, MMP2, NOTCH1, SLC2A4, KRAS, PIK3C2B, PLCG1, RFWD2 | |
| P90A0O |  | APAF1, EBF1, TP53, NF1, PLCB1, ANK2, SIRPA, ATF4, ABCC2, MMP13, BRAF, PREX1, NCOA2 | |
| 26YMUF |  | SLC2A3, TSC1, KMT2D, XDH, PLXNB1, MUC4, FZD10, ACE2, BCOR, HTR1B, SMG1, GMPS, KEAP1, KIAA1524, SRF, RICTOR | |
| DFZLO2 |  | CAPN1, PDLIM2, SERPINB2, CAMKK2, SRXN1, MAP3K1, UBA1, PLA2G4A, SLC1A5 | |
| RDD2UW | **G12D** | EXO1, ARHGEF15, PRKDC, PPP3CA, MECOM, KRAS, MAGI2, MAP4K1, TET1, EPHA3 | |
| L6ADEL |  | TP53, TRAF3 | |
| M9GYO4 |  | PTPRD, MAF, BCAR1, ZEB1, SPEN, CSF1R, MAP2K2, INSIG2, E2F1 | |
| **Nonresponders (n=18)** | | | |
| MG6XF2 |  | CTNNB1, RALGDS, EPHA5, GLS, EPHB4, DTYMK, REST, JUNB, JAK2, DAPK3, IL12RB1, SNIP1, TWIST1, MDH2 | |
| CZH5YD |  | VEGFB, PLXNB1, PTGER3, KMT2D, PTPN21, RPTOR, FGFR3, DTX1, SALL3, MYBL2, MUC4, NTN1, EPHA1, KDM6B, TGFB1I1 | |
| 3HDJMG |  | AMPD1, CAPN2, BBC3, BRCA1, ADSSL1, PC, MYLK, TET2, ARRB1, ABCG2, MSH6, LPIN1, DCLRE1C, SLC27A1 | |
| ZNT6MQ |  | SYK, NCOR2, NTRK1, MYLK, TYRO3, PDGFRA, NR1H3, BRCA1, HNF1A, MYCN, DVL1, ABCG2, SENP1, IQGAP1, KEAP1, NOS2 | |
| X0152B |  | NCOR1, ADCYAP1R1, STK11IP, SLC3A2, GLUL, TGFBR1, ANK2, TP53, CBLB, PKD1, PPFIA3, AKAP9, DDB1 | |
| QIA43T |  | BCAR1, IL18R1, RGS6, POSTN, NR3C1, RFWD2, NR1H4, OGG1, MAP2K3, ATP2A3, SMARCA4, XDH, CASP10, MAP3K11, MUC4, ATG10, PSMC6 | |
| 195P5D | **KRAS_GTP** | IKZF3, EPHB4, TP53, ERBB4, KRAS, IL4R, PPAP2B, DGKI | |
| UC2LIA |  | ANK2, TYMP, CAMKK2, ERBB4, PKD1, TCF3, SERPINE1 | |
| 67K46M |  | GATA2, EPS8, ESR1, ULK1 | |
| J0T9TJ | **G12V** | PEMT, KRAS, ROR2, IL18RAP, KMT2D, AR | |
| VCMG7N |  | ITGAM, TP53, CHEK2, MUC4, GLI3, PDIA2, ETV4, PPP2R1A | |
| L11LVL |  | HK1, PDE3A, SREBF1, TBL1X, PRKD2, FLT3, SALL3 | |
| ZX7V33 |  | TP53, SREBF1, PAK7, PIM3 | |
| GI7AGZ |  | BRAF, CES1, PPP3CA, PPP2R5E, TET3, CHN2 | |
| 3DJF3O |  | ERBB3, MUC4, EGFR, TP53, EPHB3, FOSB | |
| F3FK2W |  | MAP3K4, NF1, TIMP3, DOCK4, NFIC, TSC2, SPI1, KMT2C, PIK3CA, FANCE, BAP1, TBL1XR1 | |
| 6QFSVV | **G12C** | MYO5A, KRAS, IL21R, BARD1 | |
| IPUAS9 |  | EGFR, IGF2, HIC1 | |
| **Predictions not made (n=5)** | | | |
| 6NLFT5 |  | DGKB, ELK1, CDC73 | Insufficient information |
| 32I5VC |  |  | Insufficient information |
| IYXPLI |  | GNAQ, CES1, MUC4 | Insufficient information |
| RYRJFL |  | APBB1IP, FANCD2 | Insufficient information |
| GOFKQI |  | GNAQ, CES1, MUC4 | Insufficient information |
