# Supplementary figures and images for: Genomics of NSCLC patients both affirm PD-L1 expression and predict their clinical responses to anti-PD-1 immunotherapy
Source: BMC Cancer. 2018 Feb 27;18:225. doi: 10.1186/s12885-018-4134-y (PMC5897943; doi:10.1186/s12885-018-4134-y)

| 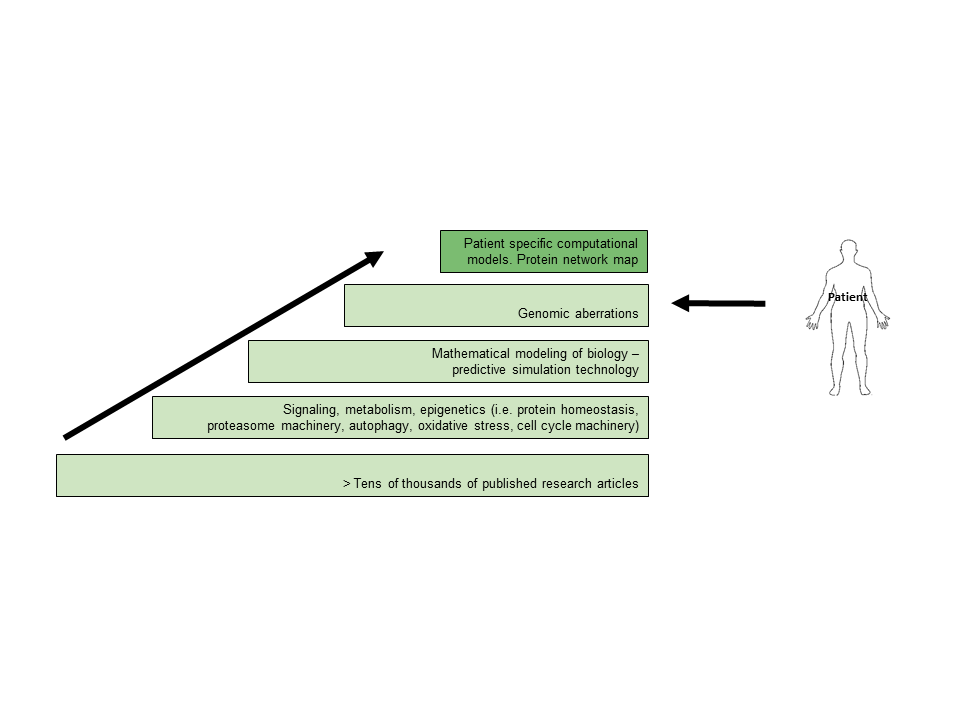 |
| --- |

Supplement: Supplementary file 3 — Figure S1. A schematic pyramid showing the levels of information used to develop the validated, cancer network. This network was created from published reports on cell receptors, signaling pathways, pathway signaling intermediates, activation factors, transcription factors, and enzyme kinetics. Information on each pathway node, its functionality, and its links with other genes, proteins, and pathways was manually researched, analyzed, curated, and aggregated to construct the integrated network maze. Every process or reaction was modeled mathematically using Michaelis Menton kinetics, mass action kinetics, and variations of these representations using ordinary differential equations (ODEs). Modeled events included but were not limited to interactions at the cell surface (e.g., binding of ligands to receptors, etc.), metabolic and cell signaling (e.g., signal pathway events, cross talk interactions among pathways, feedback control, etc.), activation and regulation of genes (e.g., activation links of transcription factors, etc.), intracellular processes such as proteasomal degradation, endoplasmic reticulum (ER) stress, oxidative stress, DNA damage and repair pathways, and cell cycle pathways. Time-dependent changes in signaling pathway fluxes of every biological reaction modeled utilizing modified ODEs were solved with a proprietary solver. Models were validated with a series of internal control analysis checks on predictions. These checks included assessing the effects of pathway molecule over-expression or knockdown on pathway predictions; effects of drugs on pathway predictions; and activation, regulation, and cross-talk interactions among pathway intermediates on pathway predictions. (DOCX 53 kb) [file 12885_2018_4134_MOESM3_ESM.docx]

| 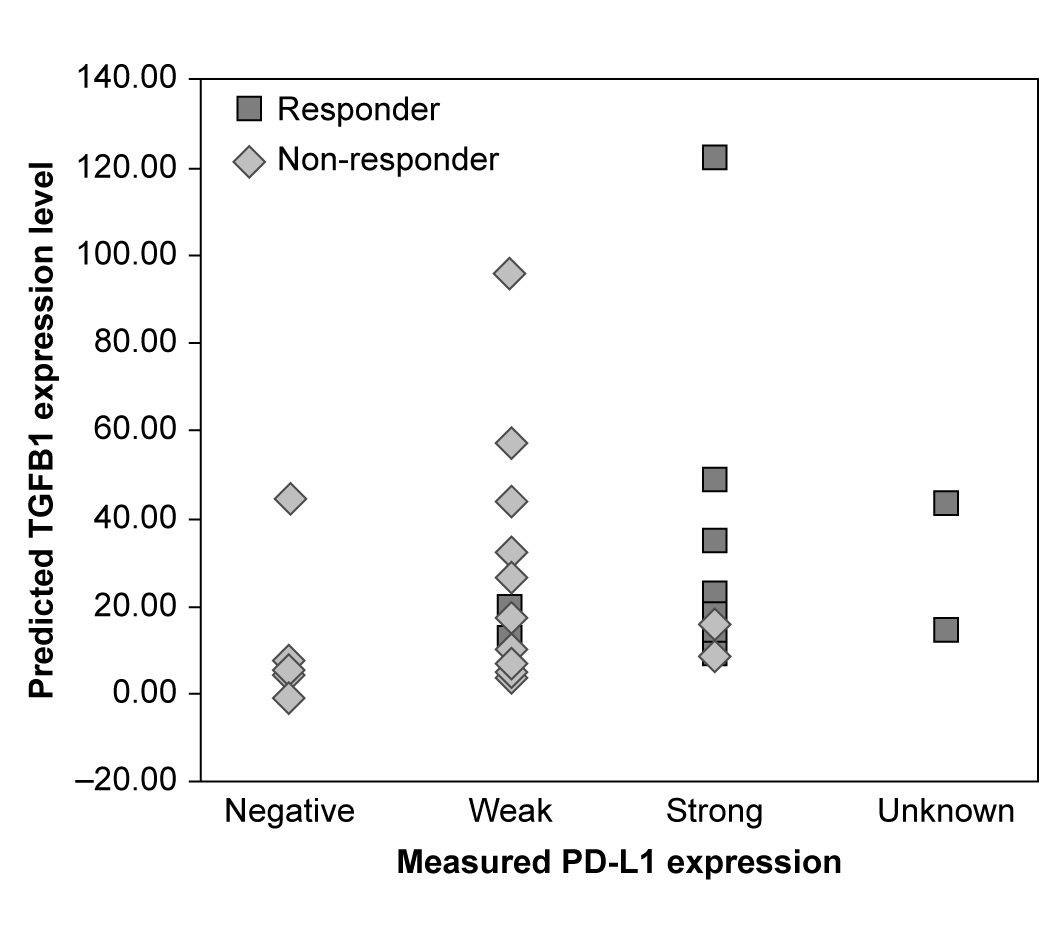 |
| --- |

Supplement: Supplementary file 8 — Figure S2. An example of the relationship between PD-L1 expression and predicted TGFB1 expression using Weka 3 algorithms for all patients in the dataset. Similar trends were seen when comparing the PD-L1 expression level to the other 13 predicted molecules. For this, the number of gene mutations identified for each patient ranged from 2 to 36 with a total of 264 unique genes between all patients. This categorical data was preprocessed and expanded into a gene vector of length 264 to represent each of the unique genes. For each gene in the vector, the data was represented in binary; a 1 was assigned if the patient had a mutation in this gene, a 0 otherwise. Two datasets, one including gene mutations (Molecules and Gene Mutations) and one without (Molecules), were both used to learn prediction models. The Discovery and Validation datasets were determined based on the split provided to allow for comparable results. The performance of a subset of these models on the testing and training sets for both Molecules and Molecules and Gene Mutations datasets are shown. The SMO support vector machine with a normalized polynomial kernel had the best performance when applied to the molecule dataset. This model correctly identified 24 out of 29 patients whereas the simulation models correctly identified 25 of 29. This was only a difference of one match between the two prediction methods. Still, several other methods, while not performing as well overall, were able to identify 9 patients in the test dataset accurately. This was near the computational simulation model prediction capability in which 10 patients were successfully identified in the test dataset. In general, adding the gene mutation data to the molecule data either maintained or decreased the performance of a model. (DOCX 4114 kb) [file 12885_2018_4134_MOESM8_ESM.docx]
