# Supplementary material for: Genomics of NSCLC patients both affirm PD-L1 expression and predict their clinical responses to anti-PD-1 immunotherapy
Source: BMC Cancer. 2018 Feb 27;18:225. doi: 10.1186/s12885-018-4134-y (PMC5897943; doi:10.1186/s12885-018-4134-y)
Supplement: Supplementary file 4 — Supplementary Materials and Methods. (DOCX 109 kb) [file 12885_2018_4134_MOESM4_ESM.docx]

**Supplementary Materials and Methods**

*Detailed description of computational model (Version 10.7)*. The simulation experiments and analysis were performed using the predictive tumor model, a comprehensive and dynamic representation of signaling and metabolic pathways in the context of cancer physiology. It provided an integrated view of all disease phenotypes. The simulation model was based on functional proteomics abstraction and enabled transparency into all internal markers with ability to run drug studies, manipulate target(s), and study impact of hypoxia and tumor microenvironment.

We adopted a bottom-up approach for building the model. The referenced current version of cancer model included more than 10,500 intracellular biological entities and ~16,000 reactions representing their interactions regulated by ~35,000 kinetic parameters. This comprised coverage of the kinome, transcriptome, proteome, and to some extent the metabolome. The simulation model included representation of signaling pathways and their complex regulatory loops such as growth factors like EGFR, PDGFR, FGFR, c-MET, VEGFR and IGF-1R, cell cycle regulators, mTOR signaling, p53 signaling cascade, apoptotic machinery, DNA damage repair, cytokine pathways like IL1, IL4, IL6, IL12, TNF; lipid mediators and tumor metabolism, oxidative and ER stress, autophagy and proteosomal representations and many others. There were 142 kinases and 102 transcription factors modeled in the system. Mathematically, we modeled the time-dependent changes in the fluxes of individual pathways using modified ordinary differential equations (ODE) and mass action kinetics.

We showed a simulation of the regulatory pathways as a demonstration of how the simulation model were developed, including the equations and parameters used. As seen in **Additional file 5**, important signaling cascades, which were effecting PD-L1 expression, were present. The network majorly flowed through activation of Ras → Raf → MEK → ERK via SHC/GRB2/SOS; activation of PI3K → AKT → mTOR signaling axis via GAB1; JAK1/2-mediated phosphorylation of STAT's that dimerized to become active transcription factors. All these signals converged to important transcription factors like NFKB, AP1, STAT3, and STAT1, which regulated PD-L1 expression.

For clarity of representation, we retained major network and cascades, which were known to impact the PD-L1 expression directly or indirectly. Modulators (like activators, inhibitors and co-activators) from other pathways were removed for the purpose of this representation. For instance, more than 10 modulators affected the activation of NFKB and STAT3/STAT1. However, for simplicity, in the snapshot we present here, we showed only few of these modulators. The representation of each of the important reaction nodes were elaborated in **Additional file 5**, which enlisted all the important reactions of the pathways contributing to PD-L1 module, the reaction equations used and literature reference for the link.

Similar to the above mentioned pathway, we developed different pathways/signalings as separate modules and integrated them with other pathways, such as HGF-Met, IGFR, PDGFR, GPCR-mediated signaling etc. The cross talks between these integrated modules included convergence of signals on common downstream effectors, feedback and feed-forward loops etc. The key differentiator of our modeling approach was its ability to represent the emergent behavior of the cell as an integrated unit, owing to the coverage and integration of various pathways and their cross talks in the same simulation.

| **Detailed description of computational model using a representative example of phosphorylation of STAT3 by mTOR signaling pathway: [STAT3 → STAT3_p → PD-L1 mRNA → PD-L1]** |
| --- |
| 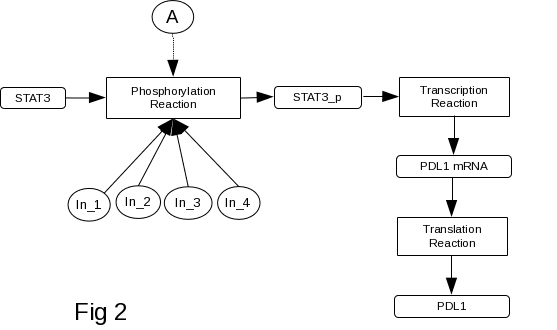 |

We modeled this reaction as a simple Michaelis-Menten equation with:

- Substrate – STAT3,
- Product1 – STAT3_p,
- Product 2 – PD-L1 (transcribed by STAT3)
- Activated by A, and
- Inhibited by 4 inhibitors – In_1, In_2, In_3, and In_4

A(mTOR) is the activated by NRAS-PIK3CA-AKT1-RHEB signaling pathway. The 4 inhibitors were:

- In_1 – PIAS3
- In_2 – SOCS1
- In_3 – SOCS3
- In_4 – STAT3 phosphorylated at Serine 727

We used the following flux equation:

(Vf_A_app * STAT3 concentration) /( Km_STAT3 + STAT3.concentration).

Vf_A_app = Vf A / {1 + (In_1 concentration/Ki_In_1)+(In_2 concentration/Ki_In_2)+(In_3 concentration/Ki_In_3)+(In_4 concentration/Ki_In_4)}

**Vf** defined the rate of the reaction. It was the product of the concentration of Kcat (rate of driving the reaction) and the concentration of activator.

**Km** was the affinity of the substrate (low Km indicating a high affinity).

**Ki** of an inhibitor was the parameter that determined the extent of inhibitory influence of the inhibitor on the reaction. Mathematically, it was the concentration of the inhibitor required to inhibit the reaction by 50%.

In the cell, the process of signaling and regulations involved protein-protein interactions and physical binding of substrate proteins and their modulators. These were represented as activations or inhibitions capturing the functional impact in the predictive simulation model. The details for the regulations modeled above were enlisted in the following table.

| **Details for the regulations modeled in the reaction** | | | |
| --- | --- | --- | --- |
| **Mechanism** | **Description** | **Parameters** | **Reference** |
| Activation  by A | mTOR mediated activation of STAT3 | Concentration of A = Dynamic*  Kcat of A = 0.419 1/sec  Vf of A = Kcat*dynamic concentration of A  Km of STAT3 = 1 uM  Concentration of STAT3 = 0.5 uM | [[1](#_ENREF_1), [2](#_ENREF_2)] |
| Inhibition  by In_1 | PIAS3 mediated inhibition of STAT3-Tyr705 phosphorylation | Concentration of PIAS3 = 0.1 uM  Ki of PIAS3 = 0.1 uM | [[3-5](#_ENREF_3)] |
| Inhibition  by In_2 | SOCS1 mediated inhibition of STAT3-Tyr705 phosphorylation | Concentration of SOCS1 = Dynamic**  Ki of SOCS1 = 19 uM | [[6-8](#_ENREF_6)] |
| Inhibition  by In_3 | SOCS3 mediated inhibition of STAT3-Tyr705 phosphorylation | Concentration of SOCS3 = Dynamic**  Ki of SOCS3 = 0.3 uM | [[9-12](#_ENREF_9)] |
| Inhibition  by In_4 | STAT3_Ser727p mediated inhibition of STAT3 -Tyr705 phosphorylation | Concentration of STAT3_Ser727p = Dynamic***  Ki of STAT3_Ser727p = 6.2 E-4 uM | [[13-16](#_ENREF_13)] |
| PD-L1 Transcription | STAT3 mediates Transcription of PD-L1 | Concentration of STAT3 = Dynamic*  Vf of A = 3.58E-2  Km of STAT3 = 1 uM | [[17](#_ENREF_17)] |
| PD-L1 Translation reaction | Synthesis of PD-L1 protein from its mRNA | Concentration of STAT3 = Dynamic*  Vf of A = 0.1  Km of STAT3 = 0.1 uM | [[17](#_ENREF_17)] |
| * Concentration of complex A (mTOR with RHEB GTP) is dynamically generated in the model when mTOR is activated by other Kinases like AKT.  ** Concentrations of In_2(SOCS1) and In_3(SOCS3) are dynamically generated as their transcription by factors like STAT1, STAT3 etc. is modeled.  *** In_4 (Ser727 phosphorylatedp form of STAT3) is dynamically generated in the model by phosphorylation of STAT3 at serine727 by kinases such as CDK1, JNK, and ERK etc.  **** PD-L1 transcription is mainly dependent on Transcription transcribing it i.e. STAT3 which is dynamically controlled by other factors mentioned above.  ***** Similarly, PD-L1 is protein synthesis is dependent on PD-L1 mRNA, which is generated dynamically from PD-L1 transcription. | | | |

Since parameters such as Kcat /Km/Ki were not well reported for signaling reactions, the parameters were reverse engineered to align to the reported end point effects in the experimental literature, which became the alignment data used.

The method of developing different integrated cell systems described above was referenced in our publications that were used in similar simulation-based approach. Importantly, we validated this simulation model extensively against prospective and retrospective *in vitro* and *in vivo* experiments.

Our simulation model aligned with NCI guidelines and emphasized robust evaluation of predictor models to determine their accuracy and completeness in addition to identifying outliers.

*Creation of patient profile for SA97V5***.** For creation of computational patient profiles first non-transformed cell models (e.g., models not containing any specific mutations and copy number variations) were used that contained integrated cancer cell networks. These networks were created from published reports on cell receptors, signaling pathways, pathway signaling intermediates, activation factors, transcription factors, and enzyme kinetics. Information on gene functionality and links between different genes, proteins, and pathways were manually researched, analyzed, curated, and aggregated to construct the integrated network maze. Reactions were modeled mathematically using Michaelis–Menten kinetics, mass action kinetics, and variations of these representations.

To start, the same non-transformed cell model was simulated until the system reached homeostatic steady state aligning it to a normal cell non-tumorigenic physiology. This established the control baseline. Patient-specific mutation profiles were annotated into the cancer physiology network model, and it was simulated to induce the profile-specific states and to predict profile-specific dysregulated pathways. The time required to achieve a network varied depending upon the complexity of the profile definition.

| **Creation of patient profile for SA97V5** |
| --- |
| 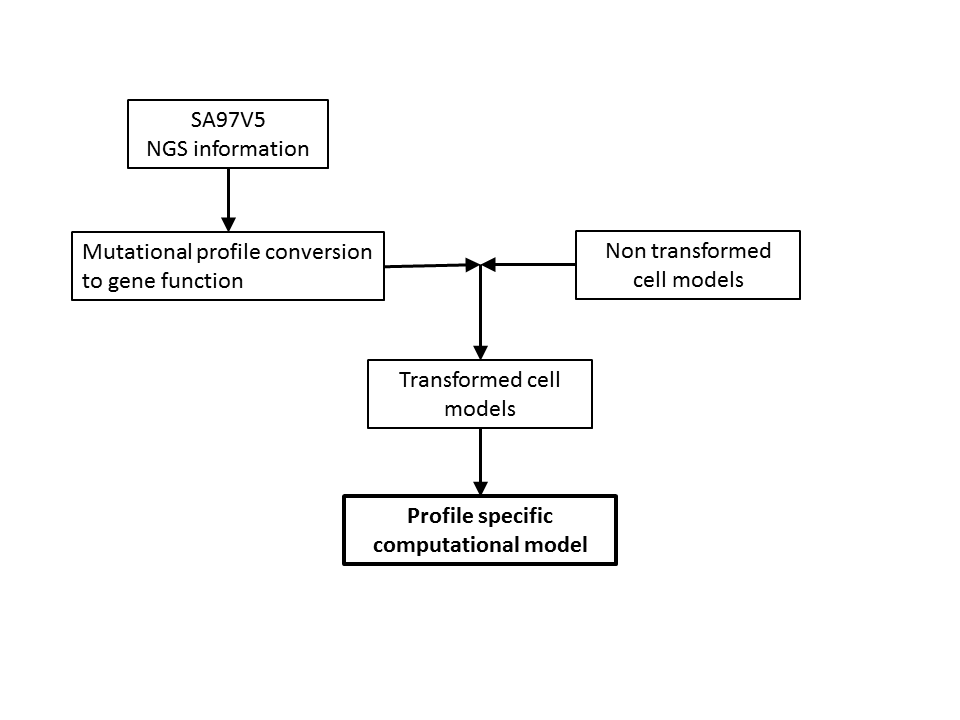 |

The SA97V5 patient-specific computational model was determined. Presence and impact of deleterious gene mutation and copy number variations on gene function were assessed. Information was imported into non-transformed predictive computational simulation models to create cell line-specific computation models.

As an example mutation reported in the patient profile SA97V5 included ANK2, TP53, AMPK, and IRS1. The simulation mutation trigger file for this cell line included the following: 1) increased parameters that define the function of ANK2 such as co-activation of growth factor receptors like KDR, IGF1R and EGFR. 2) decreased level of functional for TP53, 3) decreased level of functional for AMPK(PRKAA1), and 4) increased parameters that define the function of mutant IRS1, such as activation of Kinases like PIK3CA.

The trigger file containing mutations or copy number variations (cnv) for patient SA97V5 were present in the below mentioned table. The magnitude of perturbation of the genes in trigger file was optimized based the following factors: 1) importance and relevance of mutation/cnv for that specific cancer indication profile, 2) frequency of occurrence of mutation/cnv in that cancer indication, and 3) priority to the genomic alteration found across diverse cancer types. Patient profile-specific PD-L1 expression was reported as a percent change with respect to non-tumorigenic base-line controls and calculated within other patient profile separately. The percent change was calculated as ((D/C)-1)*100, where C is the absolute value of the non-tumorigenic base-line control (μM) and D is the absolute value of the bio-marker obtained in the cell line-specific network (μM). The same methodology was predicted for calculating percentage change for other markers also.

| **Mutations or copy number variations for patient SA97V5** | |
| --- | --- |
| **Patient ID** | **Mutations & CNV** |
| SA97V5 | TP53(DEL), TAF15(DEL), IRF9(DEL), NTRK3(DEL), PRKAA1(DEL), PTGIR(DEL), PTPN11(DEL), ITGB3(DEL), CXCR4(DEL), JAK2(DEL), BRCA2(DEL), PPP1R3A(DEL), PAX8(DEL), YWHAZ(MUT), TWIST1(MUT), ERBB3(MUT), CHN2(MUT), LCP2(MUT), SMARCA4(MUT), AMPD1(MUT), BCL2L11(MUT), RPIA(MUT), KDM5C(MUT), MED12(MUT), ATG13(MUT), APAF1(MUT), ZEB2(MUT), STAG2(MUT), SLC44A1(MUT), KMT2C(MUT), IRF8(MUT), CUL2(MUT), CREBBP(MUT), AGAP2(MUT), PREX1(MUT), IRS1(MUT), CLTC(MUT), NCOR2(MUT), UMPS(MUT), NR1H2(MUT), INSIG1(MUT), DHX38(MUT), TET1(MUT), ULK3(MUT), PPM1B(MUT), HK1(MUT), PPP1CC(MUT), RNF2(MUT), LTBP2(MUT), ITGAM(MUT), DYRK1A(MUT), CDKN2A(MUT), PPAT(MUT), HDAC6(MUT), BCR(MUT), LZTR1(MUT), RIPK4(MUT), SH3PXD2A(MUT), PIK3C2B(MUT), ABCC4(MUT), PPP1CB(MUT), PAX8(MUT), DUOX2(MUT), TOPBP1(MUT), AXIN2(MUT), ANK2(MUT), SEMA5A(MUT), TLN1(MUT), PTPN11(MUT), FASN(MUT), SRGAP1(MUT), ITGB1(MUT), CARD11(MUT), PDIA3(MUT), KDM6B(MUT), CDC42BPA(MUT), PTGER3(MUT), ROCK1(MUT), MMP16(MUT), MKNK2(MUT), |
| * CNV depicted in reddish-brown color and mutations are present in black color. | |

1. Kim JH, Yoon MS, Chen J: Signal transducer and activator of transcription 3 (STAT3) mediates amino acid inhibition of insulin signaling through serine 727 phosphorylation. J Biol Chem. 2009;284(51):35425-32.

2. Yokogami K, Wakisaka S, Avruch J, Reeves SA: Serine phosphorylation and maximal activation of STAT3 during CNTF signaling is mediated by the rapamycin target mTOR. Curr Biol. 2000;10(1):47-50.

3. Chung CD, Liao J, Liu B, Rao X, Jay P, Berta P, Shuai K: Specific inhibition of Stat3 signal transduction by PIAS3. Science. 1997;278(5344):1803-5.

4. Dabir S, Kluge A, Dowlati A: The association and nuclear translocation of the PIAS3-STAT3 complex is ligand and time dependent. Mol Cancer Res. 2009;7(11):1854-60.

5. Kluge A, Dabir S, Vlassenbroeck I, Eisenberg R, Dowlati A: Protein inhibitor of activated STAT3 expression in lung cancer. Mol Oncol. 2011;5(3):256-64.

6. Souma Y, Nishida T, Serada S, Iwahori K, Takahashi T, Fujimoto M, Ripley B, Nakajima K, Miyazaki Y, Mori M et al: Antiproliferative effect of SOCS-1 through the suppression of STAT3 and p38 MAPK activation in gastric cancer cells. Int J Cancer. 2012;131(6):1287-96.

7. Park Y, Shon SK, Kim A, Kim KI, Yang Y, Cho DH, Lee MS, Lim JS: SOCS1 induced by NDRG2 expression negatively regulates STAT3 activation in breast cancer cells. Biochem Biophys Res Commun. 2007;363(2):361-7.

8. Lee TL, Yeh J, Van Waes C, Chen Z: Epigenetic modification of SOCS-1 differentially regulates STAT3 activation in response to interleukin-6 receptor and epidermal growth factor receptor signaling through JAK and/or MEK in head and neck squamous cell carcinomas. Mol Cancer Ther. 2006;5(1):8-19.

9. Schmitz J, Weissenbach M, Haan S, Heinrich PC, Schaper F: SOCS3 exerts its inhibitory function on interleukin-6 signal transduction through the SHP2 recruitment site of gp130. J Biol Chem. 2000;275(17):12848-56.

10. Gao Y, Cimica V, Reich NC: Suppressor of cytokine signaling 3 inhibits breast tumor kinase activation of STAT3. J Biol Chem. 2012;287(25):20904-12.

11. Liang P, Cheng SH, Cheng CK, Lau KM, Lin SY, Chow EY, Chan NP, Ip RK, Wong RS, Ng MH: Platelet factor 4 induces cell apoptosis by inhibition of STAT3 via up-regulation of SOCS3 expression in multiple myeloma. Haematologica. 2013;98(2):288-95.

12. Bluyssen HA, Rastmanesh MM, Tilburgs C, Jie K, Wesseling S, Goumans MJ, Boer P, Joles JA, Braam B: IFN gamma-dependent SOCS3 expression inhibits IL-6-induced STAT3 phosphorylation and differentially affects IL-6 mediated transcriptional responses in endothelial cells. Am J Physiol Cell Physiol. 2010;299(2):C354-62.

13. Wakahara R, Kunimoto H, Tanino K, Kojima H, Inoue A, Shintaku H, Nakajima K: Phospho-Ser727 of STAT3 regulates STAT3 activity by enhancing dephosphorylation of phospho-Tyr705 largely through TC45. Genes Cells. 2012;17(2):132-45.

14. Shi X, Zhang H, Paddon H, Lee G, Cao X, Pelech S: Phosphorylation of STAT3 serine-727 by cyclin-dependent kinase 1 is critical for nocodazole-induced mitotic arrest. Biochemistry (Mosc). 2006;45(18):5857-67.

15. Lim CP, Cao X: Serine phosphorylation and negative regulation of Stat3 by JNK. J Biol Chem. 1999;274(43):31055-61.

16. Chung J, Uchida E, Grammer TC, Blenis J: STAT3 serine phosphorylation by ERK-dependent and -independent pathways negatively modulates its tyrosine phosphorylation. Mol Cell Biol. 1997;17(11):6508-16.

17. Wolfle SJ, Strebovsky J, Bartz H, Sahr A, Arnold C, Kaiser C, Dalpke AH, Heeg K: PD-L1 expression on tolerogenic APCs is controlled by STAT-3. Eur J Immunol. 2011;41(2):413-24.
