# Supplementary material for: Genomics of NSCLC patients both affirm PD-L1 expression and predict their clinical responses to anti-PD-1 immunotherapy
Source: BMC Cancer. 2018 Feb 27;18:225. doi: 10.1186/s12885-018-4134-y (PMC5897943; doi:10.1186/s12885-018-4134-y)
Supplement: Supplementary file 5 — Table S3. An example of the predictive computational modeling process. Specific details on an annexure section of the PD-L1 pathway show the step-by-step reactions, mechanisms, and reaction equations that occur. Such reactions also occurred in all of the other pathways. (DOCX 102 kb) [file 12885_2018_4134_MOESM5_ESM.docx]

| **Node No.** | **Reaction, mechanism, and reaction equation** | **References** |
| --- | --- | --- |
| **1** | Binding of EGF ligand with EGF receptor **[EGFR_f 🡪 EGFR_di]**  Binding of the ligand induces dimerization of the EGF receptor. The reaction has been modeled as a simple Michaelis-Menten equation, with the receptor as the substrate and ligand as the activator. ERKpp is an inhibitor of this reaction which is experimentally reported negative feedback loop.  (Vf_EGF*(EGFR_f.Concentration*EGFR_f.Concentration))/((Km_EGFR_f+EGFR_f.Concentration)*(Km_EGFR_f+EGFR_f.Concentration))  Where,  Vf_EGF=((kcatf_EGF*EGF_ec.Concentration)*VCyt)/ Ki_MAPK3_MAPK1_pp_app | [[1](#_ENREF_1), [2](#_ENREF_2)] |
| **2** | Autophosphorylation of the EGFR homodimer **[EGFR_di 🡪 EGFR_di_p]**  Ligand binding induces dimerization and activation of the intrinsic tyrosine kinase activity of EGFR. The reaction has been modeled as a simple Michaelis-Menten equation, which is autocatalysed.  (Vf*EGFR_di.Concentration)/((Km_EGFR_di +EGFR_di.Concentration) | [[3](#_ENREF_3)] |
| **3** | Binding & Phosphorylation of SHC1 by EGF receptor **[SHC1 🡪 SHC1_p]**  SHC1 is an adaptor protein that gets tyrosine phosphorylated by activated EGFR. The reaction has been modeled as a simple Michaelis-Menten equation with phosphorylated EGFR homodimer as an activator.  ((Vf_EGFR_di_p*SHC1.Concentration)/(Km_SHC1+SHC1.Concentration))  Where,  Vf_EGFR_di_p = (kcatf_EGFR_di_p *EGFR_di_p. Concentration)*VCyt | [[4](#_ENREF_4)] |
| **4** | Binding & Phosphorylation of GRB2 by EGF receptor **[GRB2 🡪 GRB2_p]**  The SH2 domain of GRB2 can also directly bind to phosphotyrosines 1068 and 1086 of the activated EGFR and get phosphorylated. This is in addition to SHC mediated activation of GRB2. The reaction has been modeled as a simple Michaelis-Menten equation with phosphorylated EGFR homodimer as an activator.  ((Vf_EGFR_di_p*GRB2.Concentration)/(Km_GRB2+GRB2.Concentration))  Where,  Vf_EGFR_di_p = (kcatf_EGFR_di_p *EGFR_di_p. Concentration)*VCyt | [[5](#_ENREF_5)] |
| **5** | Binding & Phosphorylation of SOS1 by GRB2 **[SOS1 🡪 SOS1_act]**  SOS1 is a Ras GEF that gets activated by GRB2p**.** This activation of SOS1 is inhibited by phosphorylated ERK. The reaction has been modeled as a simple Michaelis-Menten equation with GRB2 as an activator. The reaction flux also incorporates inhibition by activated ERK, which is a negative feedback loop in this signaling axis.  ((Vf_GRB2_p_app*SOS1.Concentration)/(Km_SOS1+SOS1.Concentration))  Where,  Vf_GRB2_p_app = (Vf_GRB2_p/(1+(MAPK3_MAPK1_pp.Concentration/Ki_MAPK3_MAPK1_pp)))  Vf_GRB2_p = (kcatf_GRB2_p*GRB2_p.Concentration)*VCyt | [[6](#_ENREF_6)] |
| **6** | Activation of RAS by EGFR activated SOS1 **[RAS_GDP 🡪 RAS_GTP]**  The reaction has been modeled as a simple Michaelis-Menten equation with SOS1 as an activator.  ((Vf_SOS1_act*RAS_GDP.Concentration)/(Km_RAS_GDP+RAS_GDP.Concentration))  Where,  Vf_SOS1_act = ((kcatf_SOS1_act*SOS1_act.Concentration)*VCyt) | [[6](#_ENREF_6)] |
| **7** | Phosphorylation of RAF by RAS-GTP **[RAF 🡪 RAF_p]**  RAS_GTP phosphorylates RAF kinase-one of its effector proteins. This reaction is inhibited by phosphorylated ERK which is a negative feedback loop of the Ras🡪Raf🡪ERK signaling axis. Additionally it is also inhibited by phosphorylated AKT which is a well reported example of cross-talks between different signaling networks in a living cell. The reaction has been modeled as a simple Michaelis-Menten equation with RAS-GTP as an activator. It also incorporates negative feedback inhibition by ERK and AKT.  ((Vf_RAS_GTP_app*RAF.Concentration)/(Km_RAF+RAF.Concentration))  Where,  Vf_RAS_GTP_app=(Vf_RAS_GTP/(1+(MAPK3_MAPK1_pp.Concentration/Ki_MAPK3_MAPK1_pp)+(AKT1_pp.Concentration/Ki_AKT1_pp))  and,  Vf_RAS_GTP= ((kcatf_RAS_GTP*RAS_GTP. Concentration)*VCyt) | [[7](#_ENREF_7)] |
| **8** | Activation of PI3K by EGFR activated GAB1 and by RAS-GTP **[PIK3CA 🡪 PIK3CA_act]**  The reaction has been modeled as a simple Michaelis-Menten equation with GAB1 as one activator and RAS_GTP as the other independent activator.  ((Vf_GAB1_P*PIK3CA.Concentration)/(Km_PIK3CA+PIK3CA.Concentration)) + ((Vf_RAS_GTP*PIK3CA.Concentration)/ (Km_PIK3CA+PIK3CA.Concentration))  Where,  Vf_GAB1_P= ((kcatf_GAB1_P*GAB1_P. Concentration)*VCyt) and Vf_RAS_GTP= ((kcatf_RAS_GTP*RAS_GTP. Concentration)*VCyt) | [[8](#_ENREF_8)] |
| **9** | Phosphorylation and activation of MEK by RAF and BRAFV600E **[MAP2K1_MAP2K2 🡪 MAP2K1_MAP2K2_p]**  The reaction has been modeled as a simple Michaelis-Menten equation with RAF as an activator.  Total=((Vf_RAF_p*MAP2K1_MAP2K2.Concentration)/(Km_MAP2K1_MAP2K2_1+MAP2K1_MAP2K2.Concentration)) + (Vf_BRAF_lV600E_di_app*MAP2K1_MAP2K2 .Concentration)/(Km_MAP2K1_MAP2K2_2+MAP2K1_MAP2K2 .Concentration)  Where,  Vf_RAF_p= ((kcatf_RAF_p*RAF_p. Concentration)*Vcyt)  Vf_BRAF_lV600E= (kcatf_BRAF_lV600E_di*BRAF_lV600E_di.Concentration)*VCyt | [[9](#_ENREF_9), [10](#_ENREF_10)] |
| **10** | Phosphorylation and activation of MEK by RAF **[MAP2K1_MAP2K2_p 🡪 MAP2K1_MAP2K2_pp]**  The reaction has been modeled as a simple Michaelis-Menten equation with RAF as an activator.  Total=((Vf_RAF_p*MAP2K1_MAP2K2_p.Concentration)/(Km_MAP2K1_MAP2K2_p+MAP2K1_MAP2K2_p.Concentration)) + + (Vf_BRAF_lV600E_di_app*MAP2K1_MAP2K2_p .Concentration)/(Km_MAP2K1_MAP2K2_p_2+MAP2K1_MAP2K2_p .Concentration)  Where,  Vf_RAF_p= ((kcatf_RAF*RAF_p. Concentration)*Vcyt)  Vf_BRAF_lV600E= (kcatf_BRAF_lV600E_di*BRAF_lV600E_di.Concentration)*VCyt | [[9](#_ENREF_9), [10](#_ENREF_10)] |
| **11** | Phosphorylation and activation of ERK by MEK **[MAPK3_MAPK1 🡪 MAPK3_MAPK1_p]**  The reaction has been modeled as a simple Michaelis-Menten equation with MEK as an activator  ((Vf_MAP2K1_MAP2K2_pp*MAPK3_MAPK1.Concentration)/(Km_MAPK3_MAPK1+MAPK3_MAPK1.Concentration))  Where,  Vf_MAP2K1_MAP2K2_pp= ((kcatf_MAP2K1_MAP2K2_pp*MAP2K1_MAP2K2_pp. Concentration)*VCyt) | [[9](#_ENREF_9)] |
| **12** | Phosphorylation and activation of ERK by MEK **[MAPK3_MAPK1_p 🡪 MAPK3_MAPK1_pp]**  The reaction has been modeled as a simple Michaelis-Menten equation with MEK as an activator  ((Vf_MAP2K1_MAP2K2_pp*MAPK3_MAPK1_p.Concentration)/(Km_MAPK3_MAPK1_p+MAPK3_MAPK1_p.Concentration))  Where,  Vf_MAP2K1_MAP2K2_pp= ((kcatf_MAP2K1_MAP2K2_pp*MAP2K1_MAP2K2_pp. Concentration)*VCyt) | [[9](#_ENREF_9)] |
| **13** | Binding & Phosphorylation of GAB1 by GRB2p **[GAB1 🡪 GAB1_p]**  Docking protein GAB1 binds phosphorylated GRB2 and gets activated. The reaction has been modeled as a simple Michaelis-Menten equation with phosphorylated GRB2 as an activator. The reaction flux also incorporates inhibition by activated ERK, which is a negative feedback loop in this signaling axis.  (Vf_GRB2_p * GAB1.Concentration)/(Km_GAB1+GAB1.Concentration))  Where,  Vf_GRB2_p = (kcatf_GRB2_p*GRB2_p.Concentration)*VCyt  and the flux is divided by the factor (1+(MAPK3_MAPK1_pp.Concentration/Ki_MAPK3_MAPK1_pp) | [[11](#_ENREF_11)] |
| **14** | PI3K mediated conversion of PIP2 to PIP3 **[PI45P2 🡪 PI345P3]**  The reaction has been modeled as a simple Michaelis-Menten equation with PI3K as an activator.  ((Vf_PIK3CA_act*PI45P2.Concentration)/(Km_PI45P2+PI45P2.Concentration))  Where,  Vf_PIK3CA_act= ((kcatf_PIK3CA_act*PIK3CA_act. Concentration)*VCyt) | [[12](#_ENREF_12)] |
| **14b** | PTEN mediated conversion of PIP3 to PIP2 **[PI345P3 🡪 PI45P2]**  The reaction has been modeled as a simple Michaelis-Menten equation with PTEN as a phosphatase (activator).  ((Vf_PTEN*PI345P3.Concentration)/(Km_PI345P3+PI345P3.Concentration))  Where,  Vf_PTEN= ((kcatf_PTEN*PTEN. Concentration)*VCyt) | [[12](#_ENREF_12)] |
| **15** | Binding of and activation PDK1 by PI345P3 **[PDPK1 🡪 PDPK1_f]**  This node is a representative of the membrane localization of PDPK1 by PIP3 binding. The reaction has been modeled as a simple Michaelis-Menten equation with PIP3 as an activator.  ((Vf_PI345P3*PDPK1.Concentration)/(Km_PDPK1+PDPK1.Concentration))  Where,  Vf_PI345P3= ((kcatf_PI345P3*PI345P3. Concentration)*VCyt) | [[12](#_ENREF_12)] |
| **16** | Binding of and activation AKT1 by PI345P3 **[AKT1_inact 🡪 AKT1]**  This node is a representative of the membrane localization of AKT1 by PIP3 binding. The reaction has been modeled as a simple Michaelis-Menten equation with PIP3 as an activator.  ((Vf_PI345P3*AKT1_inact.Concentration)/(Km_AKT1_inact+AKT1_inact.Concentration))  Where,  Vf_PI345P3= ((kcatf_PI345P3*PI345P3. Concentration)*VCyt) | [[12](#_ENREF_12)] |
| **17** | Phosphorylation of AKT1 by mTOR-Rictor on S473 **[AKT1 🡪 AKT1_p]**  mTORC2 mediated priming phosphorylation of AKT at serine 473. The reaction has been modeled as a simple Michaelis-Menten equation with mTORC2 as an activator.  ((Vf_MTOR_MAPKAP1_PRR5_MLST8_RICTOR*AKT1.Concentration)/(Km_AKT1+AKT1.Concentration))  Where,  Vf_MTOR_MAPKAP1_PRR5_MLST8_RICTOR= ((kcatf_MTOR_MAPKAP1_PRR5_MLST8_RICTOR*MTOR_MAPKAP1_PRR5_MLST8_RICTOR. Concentration)*VCyt) | [[12](#_ENREF_12)] |
| **18** | Second phosphorylation of AKT1p by PDK1 on Thr308 **[AKT1_p 🡪 AKT1_pp]**  The reaction has been modeled as a simple Michaelis-Menten equation with PDPK1 as an activator.  ((Vf_PDPK1_f*AKT1_p.Concentration)/(Km_AKT1_p+AKT1_p.Concentration))  Where,  Vf_PDPK1_f= ((kcatf_PDPK1_f*PDPK1_f. Concentration)*VCyt) | [[12](#_ENREF_12)] |
| **19** | Phosphorylation and inactivation of TSC1/2 complex by AKT **[TSC1_TSC2 🡪 TSC1_TSC2_lser939_p]**  Phosphorylation of the TSC1_TSC2 tumor suppressor complex by AKT1pp that causes its inactivation. The reaction has been modeled as a simple Michaelis-Menten equation with AKT as an activator.  ((Vf_AKT1_pp*TSC1_TSC2.Concentration)/(Km_TSC1_TSC2+TSC1_TSC2.Concentration))  Where,  Vf_AKT1_pp= ((kcatf_AKT1_pp*AKT1_pp. Concentration)*VCyt) | [[13](#_ENREF_13)] |
| **20** | Phosphorylation and activation of TSC1/2 complex by AMPK **[TSC1_TSC2 🡪 TSC1_TSC2_lthr2446_p]**  Phosphorylation of the TSC1_TSC2 tumor suppressor complex by activated AMPK that causes its activation. The reaction has been modeled as a simple Michaelis-Menten equation with AMPK as an activator.  ((Vf_PRKAA1_p*TSC1_TSC2.Concentration)/(Km_TSC1_TSC2+TSC1_TSC2.Concentration))  Where,  Vf_PRKAA1_p = ((kcatf_ PRKAA1_p * PRKAA1_p. Concentration)*VCyt) | [[14](#_ENREF_14), [15](#_ENREF_15)] |
| **21** | Dephosphorylation (inactivation) of RHEB GTP by unphosphorylated TSC complex **[RHEB_GTP 🡪 RHEB_GDP]**  The reaction has been modeled as a simple Michaelis-Menten equation with unphosphorylated TSC complex as an activator.  ((Vf_TSC1_TSC2*RHEB_GTP.Concentration)/(Km_RHEB_GTP+RHEB_GTP.Concentration))  Where,  Vf_TSC1_TSC2= ((kcatf_TSC1_TSC2*TSC1_TSC2. Concentration)*VCyt) | [[16](#_ENREF_16)] |
| **22** | Dephosphorylation (inactivation) of RHEB GTP by TSC complex phosphorylated by AMPK **[RHEB_GTP 🡪 RHEB_GDP]**  The reaction has been modeled as a simple Michaelis-Menten equation with Thr phosphorylated TSC complex as an activator.  ((Vf_TSC1_TSC2_lthr2446_p*RHEB_GTP.Concentration)/(Km_RHEB_GTP+RHEB_GTP.Concentration))  Where,  Vf_TSC1_TSC2_lthr2446_p= ((kcatf_TSC1_TSC2_lthr2446_p*TSC1_TSC2_lthr2446_p. Concentration)*VCyt) | [[16](#_ENREF_16)] |
| **23** | Conversion of RHEB GDP to RHEB GTP**[RHEB_GDP 🡪 RHEB_GTP]**  The reaction has been modeled as a simple Michaelis-Menten equation.  ((Vf*RHEB_GDP.Concentration)/(Km_RHEB_GDP+RHEB_GDP.Concentration)) | [[16](#_ENREF_16)] |
| **24** | Activation of mTOR complex **[MLST8_MTOR_RPTOR 🡪 MTOR_RPTOR_MLST8_RHEB_GTP**]  The reaction has been modeled as a simple Michaelis-Menten equation with RHEB-GTP as an activator.  ((Vf_RHEB_GTP*MLST8_MTOR_RPTOR.Concentration)/(Km_MLST8_MTOR_RPTOR+MLST8_MTOR_RPTOR.Concentration))  Where,  Vf_RHEB_GTP= ((kcatf_RHEB_GTP*RHEB_GTP. Concentration)*VCyt) | [[16](#_ENREF_16)] |
| **25** | Phosphorylation of PLC gamma by the EGF receptor **[PLCG1🡪 PLCG1_p]**  The reaction has been modeled as a simple Michaelis-Menten equation with EGFR as an activator.  (Vf_EGFR_di_p*PLCG1.Concentration)/(Km_PLCG1+PLCG1.Concentration)  Where,  Vf_ EGFR_di_p = ((kcatf_EGFR_di_p*EGFR_di_p.Concentration)*VCyt) | [[17](#_ENREF_17)] |
| **26** | PIP2 Hydrolysis by PLC gamma **[PI45P2 🡪 DAG + IP3]**  The reaction has been modeled as a simple Michaelis-Menten equation with PLCG as an activator and PIP2 as the substrate.  (Vf_PLCG1_p*PI45P2.Concentration)/(Km_PI45P2+PI45P2.Concentration)  Where,  Vf_PLCG1_p = (kcatf_PLCG1_p*PLCG1_p.Concentration)*VCyt | [[18](#_ENREF_18)] |
| **27** | Binding of PKC-Calcium complex with DAG **[PRKCA_p_Ca 🡪 PRKCA_p_Ca_DAG]**  The reaction has been modeled as a simple Michaelis-Menten equation with DAG as an activator and PKC Calcium complex as the substrate.  (Vf_DAG*PRKCA_p_Ca.Concentration)/(Km_PRKCAp_ca_c+PRKCA_p_Ca.Concentration)  Where,  Vf_DAG = ((DAG.Concentration*kcatf_DAG)*VCyt) | [[19](#_ENREF_19)] |
| **28** | Binding & Phosphorylation of JAK1 by EGF receptor **[JAK1🡪 EGFR_di_p_JAK1_p]**  The reaction has been modeled as a simple Michaelis-Menten equation with EGFR as an activator.  (Vf_EGFR_di_p*JAK1.Concentration)/(Km_JAK1+JAK1.Concentration) | [[20](#_ENREF_20)] |
| **29** | Binding & Phosphorylation of JAK2 by EGF receptor activated JAK1 complex **[JAK2 🡪 EGFR_di_p_JAK1_p_JAK2_p]**  The reaction has been modeled as a simple Michaelis-Menten equation with EGFR_JAK1p complex as an activator.  (Vf_EGFR_di_p_JAK1_p *JAK2.Concentration)/(Km_JAK2+JAK2.Concentration) | [[20](#_ENREF_20)] |
| **30** | Phosphorylation of STAT1 by the EGF receptor activated JAK1-JAK2 complex **[STAT1 🡪 STAT1_p]**  The reaction has been modeled as a simple Michaelis-Menten equation with EGFR_JAK1_JAK2 complex as an activator and incorporates inhibition by SOCS1, SOCS3 and PIAS4.  (Vf_EGFR_di_p_JAK1_p_JAK2_p*STAT1.Concentration)/(Km_STAT1+STAT1.Concentration)  Where,  Vf_EGFR_di_p_JAK1_p_JAK2_p = (kcatf_EGFR_di_p_JAK1_p_JAK2_p*EGFR_Cyt.EGFR_di_p_JAK1_p_JAK2_p.Concentration)*VCyt/Ki_PIAS4_app/Ki_SOCS1_app/Ki_SOCS3_app | [[21](#_ENREF_21)] |
| **31** | Acetylation of STAT3 **[STAT3 🡪 STAT3_ac]**  The reaction has been modeled as a simple Michaelis-Menten equation with CREBBP as acetylating agent.  ((Vf_CREBBP_p_act_n_app*STAT3.Concentration)/(Km_STAT3_1+STAT3.Concentration))  Where Vf_CREBBP_p_act_n= (kcatf_CREBBP_p_act_n*N.CREBBP_p_act_n.Concentration)*Vcyt  &  kcatf_CREBBP_p_act_n=37.7 1/sec | [[22](#_ENREF_22)] |
| **32** | Phosphorylation of STAT3 by the EGF receptor activated JAK1-JAK2 complex **[STAT3_ac 🡪 STAT3_ac_p]**  The reaction has been modeled as a simple Michaelis-Menten equation with EGFR_JAK1_JAK2 complex as an activator and incorporates inhibition by SOCS1, SOCS3 and PIAS3.  (Vf_EGFR_di_p_JAK1_p_JAK2_p_app*STAT3.Concentration)/(Km_STAT3 +STAT3.Concentration)  Where,  Vf_EGFR_di_p_JAK1_p_JAK2_p_app = (Vf_EGFR2P_JAK1_JAK2p/(1+(PIAS3.Concentration/Ki_PIAS3)))/Ki_STAT3_lSer727_p_app/Ki_SOCS1_app/Ki_SOCS3_app | [[20](#_ENREF_20), [21](#_ENREF_21)] |
| **33** | Dimerization of phosphorylated STAT3 **[STAT3_ac 🡪 STAT3_p_ac_di]**  The reaction has been modeled as a simple Michaelis-Menten equation.  (Vf*STAT3_ac_p.Concentration)/(Km_STAT3_ac_p+STAT3_ac_p.Concentration) | [[23](#_ENREF_23)] |
| **34** | Ser 727 Phosphorylation of STAT3 by the mTOR complex **[STAT3 🡪 STAT3_Ser727_p]**  The reaction has been modeled as a simple Michaelis-Menten equation.  (Vf_MTOR_RPTOR_MLST8_RHEB_GTP_app*STAT3.Concentration)/(Km_STAT3+STAT3.Concentration) | [[24](#_ENREF_24)] |
| **35** | Dimerization of phosphorylated STAT3 **[STAT3_Ser727_p 🡪 STAT3_Ser727p_di]**  The reaction has been modeled as a simple Michaelis-Menten equation.  (Vf*STAT3_lSer727_p.Concentration)/(Km_STAT3_lSer727_p+STAT3_lSer727_p.Concentration) | [[25](#_ENREF_25)] |
| **36** | IFNGR2 in bound form with JAK2 **[IFNGR2+JAK2 🡪 IFNGR2_JAK2 ]**  The reaction has been modeled as a simple Michaelis-Menten equation.  (Vf_app*JAK2.Concentration)/(Km_JAK2+JAK2.Concentration)  Where  Vf_app= kcatf_IFNGR2*IFNGR2.Concentration*VCyt | [[26](#_ENREF_26)] |
| **37** | IFNGR1 in bound form with JAK1 **[IFNGR1+JAK1 🡪 IFNGR1_JAK1 ]**  The reaction has been modeled as a simple Michaelis-Menten equation.  (Vf_app*JAK2.Concentration)/(Km_JAK2+JAK2.Concentration)  Where  Vf_app= kcatf_IFNGR1*IFNGR1.Concentration*VCyt | [[26](#_ENREF_26)] |
| **38** | Binding IFN-gamma to the complex IFNGR1_JAK1 **[IFNGR1_JAK1 🡪 IFNGR1_JAK1_act]**  The reaction has been modeled as a simple Michaelis-Menten equation.  (Vf_IFNG_ec*IFNGR1_JAK1.Concentration)/(Km_IFNGR1_JAK1+IFNGR1_JAK1.Concentration) | [[26](#_ENREF_26)] |
| **39** | Binding of IFNGR1_JAK1_IFNG to the complex IFNGR2_JAK2 **[IFNGR2_JAK2 🡪 IFNGR2_JAK2_act]**  The reaction has been modeled as a simple Michaelis-Menten equation.  (Vf_IFNGR1_JAK1_act*IFNGR2_JAK2.Concentration)/(Km_IFNGR2_JAK2+IFNGR2_JAK2.Concentration) | [[26](#_ENREF_26)] |
| **40** | Phosphorylation of STAT1 by IFNGR and JAK complex **[STAT1 🡪 STAT1_p]**  The reaction has been modeled as a simple Michaelis-Menten equation with IFNG_JAK1_JAK2 complex as an activator and incorporates inhibition by SOCS1, SOCS3 and PIAS4.  (Vf_IFNGR2_JAK2_act*STAT1.Concentration)/(Km_STAT1_2+STAT1.Concentration) +  Where,  Vf_IFNGR2_JAK2_act=  (kcatf_IFNGR2_JAK2_act*IFNGR2_JAK2_act .Concentration)*VCyt/Ki_PIAS4_app/Ki_SOCS1_app/Ki_SOCS3_app | [[21](#_ENREF_21)]  [[27](#_ENREF_27)] |
| **41** | Dimerization of phosphorylated STAT1 **[STAT1_p 🡪 STAT1_p_di]**  The reaction has been modeled as a simple Michaelis-Menten equation.  (Vf*STAT1_p.Concentration)/(Km_STAT1_p+STAT1_p.Concentration) | [[28](#_ENREF_28)] |
| **42** | Transcription of PDL1 by STAT3 , Ser phosphorylated STAT3 and STAT1 **[STAT3 🡪 PDL1 mRNA]**  The reaction has been modeled as a simple Michaelis-Menten equation with STAT3, STAt1 in nucleus as Transcription factor with AP1 as co-activator and DNMT/miRNA as inhibitors.  Total=((Vf_STAT3_p_ac_di_n_app*STAT3_p_ac_di_n.Concentration)/(Km_STAT3_p_ac_di_n_app+STAT3_p_ac_di_n.Concentration)) + (Vf_STAT3_lSer727_p_di_n_app*STAT3_lSer727_p_di_n.Concentration)/(Km_STAT3_lSer727_p_di_n_app+STAT3_lSer727_p_di_n.Concentration)  + (Vf_STAT1_p_di_n_app*STAT1_p_di_n.Concentration)/(Km_STAT1_p_di_n_app+STAT1_p_di_n.Concentration)  where Vf_STAT3_p_ac_di_n_app = ((((((Vf_STAT3_p_ac_di_n*Ka_FOS_p_JUN_p_ac_n_app)/Ki_DNMT3A_act_DNMT3B_act_CpGDNA_m_n_app))/ICF_MIR34A_n))/ICF_MIR200C_n)  Vf_STAT3_lSer727_p_di_n= ((((((Vf_STAT3_lSer727_p_di_n*Ka_FOS_p_JUN_p_ac_n_app)/Ki_DNMT3A_act_DNMT3B_act_CpGDNA_m_n_app))/ICF_MIR34A_n))/ICF_MIR200C_n)  Vf_STAT1_p_di_n_app= ((((((Vf_STAT1_p_di_n*Ka_FOS_p_JUN_p_ac_n_app)/Ki_DNMT3A_act_DNMT3B_act_CpGDNA_m_n_app))/ICF_MIR34A_n))/ICF_MIR200C_n) | [[29](#_ENREF_29)] |
| **43** | Translation of PDL1 **[PDL1 mRNA 🡪 PDL1]**  The reaction has been modeled as a simple Michaelis-Menten equation  (Vf_CD274_mRNA*CD274_mRNA.Concentration)/(Km_CD274_mRNA_app+CD274_mRNA.Concentration) |  |

1. Moriki T, Maruyama H, Maruyama IN: Activation of preformed EGF receptor dimers by ligand-induced rotation of the transmembrane domain. J Mol Biol. 2001;311(5):1011-26.

2. Turke AB, Song Y, Costa C, Cook R, Arteaga CL, Asara JM, Engelman JA: MEK inhibition leads to PI3K/AKT activation by relieving a negative feedback on ERBB receptors. Cancer Res. 2012;72(13):3228-37.

3. Yu X, Sharma KD, Takahashi T, Iwamoto R, Mekada E: Ligand-independent dimer formation of epidermal growth factor receptor (EGFR) is a step separable from ligand-induced EGFR signaling. Mol Biol Cell. 2002;13(7):2547-57.

4. Sakaguchi K, Okabayashi Y, Kido Y, Kimura S, Matsumura Y, Inushima K, Kasuga M: Shc phosphotyrosine-binding domain dominantly interacts with epidermal growth factor receptors and mediates Ras activation in intact cells. Mol Endocrinol. 1998;12(4):536-43.

5. Okutani T, Okabayashi Y, Kido Y, Sugimoto Y, Sakaguchi K, Matuoka K, Takenawa T, Kasuga M: Grb2/Ash binds directly to tyrosines 1068 and 1086 and indirectly to tyrosine 1148 of activated human epidermal growth factor receptors in intact cells. J Biol Chem. 1994;269(49):31310-4.

6. Langlois WJ, Sasaoka T, Saltiel AR, Olefsky JM: Negative feedback regulation and desensitization of insulin- and epidermal growth factor-stimulated p21ras activation. J Biol Chem. 1995;270(43):25320-3.

7. Hallberg B, Rayter SI, Downward J: Interaction of Ras and Raf in intact mammalian cells upon extracellular stimulation. J Biol Chem. 1994;269(6):3913-6.

8. Mattoon DR, Lamothe B, Lax I, Schlessinger J: The docking protein Gab1 is the primary mediator of EGF-stimulated activation of the PI-3K/Akt cell survival pathway. BMC Biol. 2004;2:24.

9. Gardner AM, Vaillancourt RR, Lange-Carter CA, Johnson GL: MEK-1 phosphorylation by MEK kinase, Raf, and mitogen-activated protein kinase: analysis of phosphopeptides and regulation of activity. Mol Biol Cell. 1994;5(2):193-201.

10. Joseph EW, Pratilas CA, Poulikakos PI, Tadi M, Wang W, Taylor BS, Halilovic E, Persaud Y, Xing F, Viale A et al: The RAF inhibitor PLX4032 inhibits ERK signaling and tumor cell proliferation in a V600E BRAF-selective manner. Proc Natl Acad Sci U S A. 2010;107(33):14903-8.

11. Bonderman RP, Bonderman DP: Atypical and inhibited human serum pseudocholinesterase. A titrimetric method for differentiation. Arch Environ Health. 1971;22(5):578-81.

12. Nicholson KM, Anderson NG: The protein kinase B/Akt signalling pathway in human malignancy. Cell Signal. 2002;14(5):381-95.

13. Tee AR, Anjum R, Blenis J: Inactivation of the tuberous sclerosis complex-1 and -2 gene products occurs by phosphoinositide 3-kinase/Akt-dependent and -independent phosphorylation of tuberin. J Biol Chem. 2003;278(39):37288-96.

14. Gwinn DM, Shackelford DB, Egan DF, Mihaylova MM, Mery A, Vasquez DS, Turk BE, Shaw RJ: AMPK phosphorylation of raptor mediates a metabolic checkpoint. Mol Cell. 2008;30(2):214-26.

15. Huang J, Manning BD: The TSC1-TSC2 complex: a molecular switchboard controlling cell growth. Biochem J. 2008;412(2):179-90.

16. Long X, Lin Y, Ortiz-Vega S, Yonezawa K, Avruch J: Rheb binds and regulates the mTOR kinase. Curr Biol. 2005;15(8):702-13.

17. Chattopadhyay A, Vecchi M, Ji Q, Mernaugh R, Carpenter G: The role of individual SH2 domains in mediating association of phospholipase C-gamma1 with the activated EGF receptor. J Biol Chem. 1999;274(37):26091-7.

18. Hao JJ, Liu Y, Kruhlak M, Debell KE, Rellahan BL, Shaw S: Phospholipase C-mediated hydrolysis of PIP2 releases ERM proteins from lymphocyte membrane. J Cell Biol. 2009;184(3):451-62.

19. Reither G, Schaefer M, Lipp P: PKCalpha: a versatile key for decoding the cellular calcium toolkit. J Cell Biol. 2006;174(4):521-33.

20. Park OK, Schaefer TS, Nathans D: In vitro activation of Stat3 by epidermal growth factor receptor kinase. Proc Natl Acad Sci U S A. 1996;93(24):13704-8.

21. Ihle JN, Nosaka T, Thierfelder W, Quelle FW, Shimoda K: Jaks and Stats in cytokine signaling. Stem Cells. 1997;15 Suppl 1:105-11; discussion 12.

22. Zhang Y, Sif S, DeWille J: The mouse C/EBPdelta gene promoter is regulated by STAT3 and Sp1 transcriptional activators, chromatin remodeling and c-Myc repression. J Cell Biochem. 2007;102(5):1256-70.

23. Mitchell TJ, John S: Signal transducer and activator of transcription (STAT) signalling and T-cell lymphomas. Immunology. 2005;114(3):301-12.

24. Yokogami K, Wakisaka S, Avruch J, Reeves SA: Serine phosphorylation and maximal activation of STAT3 during CNTF signaling is mediated by the rapamycin target mTOR. Curr Biol. 2000;10(1):47-50.

25. Sakaguchi M, Oka M, Iwasaki T, Fukami Y, Nishigori C: Role and regulation of STAT3 phosphorylation at Ser727 in melanocytes and melanoma cells. J Invest Dermatol. 2012;132(7):1877-85.

26. Woldman I, Varinou L, Ramsauer K, Rapp B, Decker T: The Stat1 binding motif of the interferon-gamma receptor is sufficient to mediate Stat5 activation and its repression by SOCS3. J Biol Chem. 2001;276(49):45722-8.

27. Li N, McLaren JE, Michael DR, Clement M, Fielding CA, Ramji DP: ERK is integral to the IFN-gamma-mediated activation of STAT1, the expression of key genes implicated in atherosclerosis, and the uptake of modified lipoproteins by human macrophages. J Immunol. 2010;185(5):3041-8.

28. Harvey EJ, Li N, Ramji DP: Critical role for casein kinase 2 and phosphoinositide-3-kinase in the interferon-gamma-induced expression of monocyte chemoattractant protein-1 and other key genes implicated in atherosclerosis. Arterioscler Thromb Vasc Biol. 2007;27(4):806-12.

29. Wolfle SJ, Strebovsky J, Bartz H, Sahr A, Arnold C, Kaiser C, Dalpke AH, Heeg K: PD-L1 expression on tolerogenic APCs is controlled by STAT-3. Eur J Immunol. 2011;41(2):413-24.
