# Supplementary material for: Genomics of NSCLC patients both affirm PD-L1 expression and predict their clinical responses to anti-PD-1 immunotherapy
Source: BMC Cancer. 2018 Feb 27;18:225. doi: 10.1186/s12885-018-4134-y (PMC5897943; doi:10.1186/s12885-018-4134-y)
Supplement: Supplementary file 6 — Table S4. Creation of the dendritic cell infiltration index for the patient SA97V5-specific simulation model. Chemokines CCL11, CCL20, CCL2, CCL3, CCL4, CCL5, CCL7, CX3CL1, and CXCL14, capable of trafficking of dendritic cells into the tumor microenvironment, were used to create the index. Individual chemokine percent expression (with respect to non-tumorigenic baseline controls) was predicted and given weightage so as to normalize the total to 1. The index was then calculated to be the sum of each prediction % change * weightage. (DOCX 16 kb) [file 12885_2018_4134_MOESM6_ESM.docx]

| **S.NO** | **Chemokine** | **Predicted Baseline Value (uM)**  **C** | **Predicted Disease Value (uM)**  **D** | **% Change**  **= ( (D-C)/C)*100**  **P** | **Weightage for**  **Dendritic cell infiltration index** | **% Contribution of each chemokine in**  **DC Infiltration index**  **= (P*weightage)** |
| --- | --- | --- | --- | --- | --- | --- |
| 1 | CCL11 | 7.32056E-07 | 1.00188E-06 | 36.86 | 0.067 | 2.47 |
| 2 | CCL20 | 5.80529E-06 | 7.58137E-06 | 30.59 | 0.067 | 2.05 |
| 3 | CCL2 | 1.68183E-07 | 2.16436E-07 | 28.69 | 0.200 | 5.74 |
| 4 | CCL3 | 9.26144E-08 | 1.05862E-07 | 14.30 | 0.067 | 0.96 |
| 5 | CCL4 | 1.37827E-05 | 1.75323E-05 | 27.21 | 0.200 | 5.44 |
| 6 | CCL5 | 0.000145274 | 0.000164734 | 13.39 | 0.200 | 2.68 |
| 7 | CCL7 | 8.68126E-06 | 1.19807E-05 | 38.01 | 0.067 | 2.55 |
| 8 | CX3CL1 | 1.03246E-05 | 1.37213E-05 | 32.90 | 0.067 | 2.20 |
| 9 | CXCL14 | 1.17089E-06 | 1.13287E-06 | -3.25 | 0.067 | -0.22 |
| **Dendritic cell infiltration index** | | | | | | **23.87** |

C_Value = Starting absolute value of chemokine.

D_Value = Final absolute value of chemokine obtained after creation of the patient SA97V5-specific simulation model.

Weightage = Weightage given to each chemokine so as to normalize the total to 1.
