# Supplementary material for: Genomics of NSCLC patients both affirm PD-L1 expression and predict their clinical responses to anti-PD-1 immunotherapy
Source: BMC Cancer. 2018 Feb 27;18:225. doi: 10.1186/s12885-018-4134-y (PMC5897943; doi:10.1186/s12885-018-4134-y)
Supplement: Supplementary file 7 — Table S5. Analysis of the Discovery and Validation datasets was performed using Weka 3. The first number in each column represented the number of patient treatment responses correctly classified by the model. The second number represented the number of incorrectly classified patient treatment responses. The GOAL row at the bottom of each column described the number of correctly and incorrectly classified patients in the simulation models. The Test Set columns described the output from applying the model trained on the Discovery set to the Validation set. The “Test and Train” columns described test set accuracy (test set column) plus the training error (results obtained by applying the model to the training set, i.e. training error). (DOCX 19 kb) [file 12885_2018_4134_MOESM7_ESM.docx]

| **Prediction:**  **(# match, #mismatch)** | **Test Set Molecules** | **Test and Train Molecules** | **Test Set Molecule and Gene Mutations** | **Test and Train Molecule and Gene Mutations** |
| --- | --- | --- | --- | --- |
| ZeroR^a^ | 7,4 | 18,11 | 7,4 | 18,11 |
| Logistic Regression^b^ | 7,4 | 24,5 | 6,5 | 18,11 |
| Naïve Bayes^c^ | 6,5 | 16,13 | 6,5 | 18,11 |
| RBFNetwork^d^ | 9,2 | 23,6 | 4,7 | 16,13 |
| ADTree^e^ | 9,2 | 19,10 | 9,2 | 19,10 |
| BFTree^f^ | 9,2 | 20,9 | 9,2 | 20,9 |
| J48 Tree^g^ | 9,2 | 19,10 | 9,2 | 20,9 |
| SMO^h^ | 7,4 | 23,6 | 6,5 | 20,9 |
| SMO with NormPolyKern^i^ | 8,3 | 24,5 | 6,5 | 18,11 |
| Voted Perceptron^j^ | 9,2 | 19,10 | 9,2 | 19,10 |
| Multilayer Perceptron^k^ | 7,4 | 22,7 | 6,5 | 21,8 |
| GOAL | 10,1 | 25,4 | 10,1 | 25,4 |
| ^a^weka.classifiers.rules.ZeroR  ^b^weka.classifiers.functions.Logistic -R 1.0E-8 -M -1  ^c^weka.classifiers.bayes.NaiveBayes  ^d^weka.classifiers.functions.RBFNetwork -B 2 -S 1 -R 1.0E-8 -M -1 -W 0.1  ^e^weka.classifiers.trees.ADTree -B 10 -E -3  ^f^weka.classifiers.trees.BFTree -S 1 -M 2 -N 5 -C 1.0 -P POSTPRUNED  ^g^weka.classifiers.trees.J48 -C 0.25 -M 2  ^h^weka.classifiers.functions.SMO -C 1.0 -L 0.001 -P 1.0E-12 -N 0 -V -1 -W 1 -K  "weka.classifiers.functions.supportVector.PolyKernel -C 250007 -E 1.0"  ^i^weka.classifiers.functions.SMO -C 1.0 -L 0.001 -P 1.0E-12 -N 0 -V -1 -W 1 -K  "weka.classifiers.functions.supportVector.NormalizedPolyKernel -C 250007 -E 2.0"  ^j^weka.classifiers.functions.Voted Perceptron -I 1 -E 1.0 -S 1 -M 10000  ^k^weka.classifiers.functions.MultilayerPerceptron -L 0.3 -M 0.2 -N 500 -V 0 -S 0 -E 20 -H a | | | | |
