# Supplementary material for: Genomics of NSCLC patients both affirm PD-L1 expression and predict their clinical responses to anti-PD-1 immunotherapy
Source: BMC Cancer. 2018 Feb 27;18:225. doi: 10.1186/s12885-018-4134-y (PMC5897943; doi:10.1186/s12885-018-4134-y)
Supplement: Supplementary file 9 — Table S6. Comparisons of clinical and predicated responses and match scores. We used a cross-validation approach to assess the match scores in Table 1 of the PD-1 predicted responses against the PD-1 clinical responses in the Rizvi et al. 2015 Discovery dataset vs. the Validation dataset. We then pooled and re-partitioned the dataset into two new Training and Test datasets. We then used a similar cross-validation approach to assess the match scores of the PD-1 predicted responses vs. the PD-1 clinical responses. (DOCX 17 kb) [file 12885_2018_4134_MOESM9_ESM.docx]

| Comparisons of clinical and predicated responses and match score between Discovery and Validation groups | | | |
| --- | --- | --- | --- |
| **Outcome** | **Discovery Group**  **(N=13)**  **n (%)** | **Validation Group**  **(N=16)**  **n (%)** | **P-value** |
| **Clinical Response**  Non-Responder  Responder | 8 (61.5)  5 (38.5) | 10 (62.5)  6 (37.5) | 0.9577* |
| **Predicted Response**  Non-Responder  Responder | 9 (69.2)  4 (30.8) | 7 (43.8)  9 (56.2) | 0.2642* |
| **Score**  Match  Mismatch | 12 (92.3)  1 (7.7) | 13 (81.2)  3 (18.8) | 0.6059* |
| Comparisons of clinical and predicted responses and match rates between Training and Test groups. | | | |
| **Outcome** | **Training Group (N=18)**  **n (%)** | **Test Group**  **(N=11)**  **n (%)** | **P-value** |
| **Clinical Response**  Non-Responder  Responder | 11 (61.1)  7 (38.9) | 7 (63.6)  4 (36.4) | 0.9999* |
| **Predicted Response**  Non-Responder  Responder | 10 (55.6)  8 (44.4) | 6 (54.5)  5 (45.5) | 0.9577* |
| **Score**  Match  Mismatch | 15 (83.3)  3 (16.7) | 10 (90.9)  1 (9.1) | 0.9999* |

*****P>0.05 considered statistically non-significant using the chi-square test or Fisher’s exact test
